# Killer whale (*Orcinus orca*) population dynamics in response to a period of rapid ecosystem change in the eastern North Atlantic

**DOI:** 10.1002/ece3.8364

**Published:** 2021-11-18

**Authors:** Eve Jourdain, Tiffany Goh, Sanna Kuningas, Tiu Similä, Dag Vongraven, Richard Karoliussen, Anna Bisther, Philip S. Hammond

**Affiliations:** ^1^ Norwegian Orca Survey Andenes Norway; ^2^ Department of Biosciences University of Oslo Oslo Norway; ^3^ Sea Mammal Research Unit Scottish Oceans Institute University of St Andrews Fife UK; ^4^ Natural Resources Institute Finland Helsinki Finland; ^5^ Whale2Sea Andenes Norway; ^6^ Norwegian Polar Institute Tromsø Norway; ^7^ Reportagebörsen Gothenburg Sweden

**Keywords:** abundance, apparent survival, capture heterogeneity, killer whale, photo‐identification, population dynamics

## Abstract

This study investigates survival and abundance of killer whales (*Orcinus orca*) in Norway in 1988–2019 using capture–recapture models of photo‐identification data. We merged two datasets collected in a restricted fjord system in 1988–2008 (Period 1) with a third, collected after their preferred herring prey shifted its wintering grounds to more exposed coastal waters in 2012–2019 (Period 2), and investigated any differences between these two periods. The resulting dataset, spanning 32 years, comprised 3284 captures of 1236 whales, including 148 individuals seen in both periods. The best‐supported models of survival included the effects of sex and time period, and the presence of transients (whales seen only once). Period 2 had a much larger percentage of transients compared to Period 1 (mean = 30% vs. 5%) and the identification of two groups of whales with different residency patterns revealed heterogeneity in recapture probabilities. This caused estimates of survival rates to be biased downward (females: 0.955 ± 0.027 SE, males: 0.864 ± 0.038 SE) compared to Period 1 (females: 0.998 ± 0.002 SE, males: 0.985 ± 0.009 SE). Accounting for this heterogeneity resulted in estimates of apparent survival close to unity for regularly seen whales in Period 2. A robust design model for Period 2 further supported random temporary emigration at an estimated annual probability of 0.148 (± 0.095 SE). This same model estimated a peak in annual abundance in 2015 at 1061 individuals (95% CI 999–1127), compared to a maximum of 731 (95% CI 505–1059) previously estimated in Period 1, and dropped to 513 (95% CI 488–540) in 2018. Our results indicate variations in the proportion of killer whales present of an undefined population (or populations) in a larger geographical region. Killer whales have adjusted their distribution to shifts in key prey resources, indicating potential to adapt to rapidly changing marine ecosystems.

## Graphical abstract

Mark–recapture models fitted to a 32‐year photo‐identification dataset suggest constant, high apparent survival rates for killer whales in Norway but reveal temporal variations in abundance and movement patterns in response to a period of ecosystem change. These results reflected variations in the proportion present of an undefined population (or populations) in a larger geographical region of the north‐eastern Atlantic. Our results show that killer whales have adjusted their distribution to shifts in key prey resources, indicating potential to adapt to rapidly changing marine ecosystems.

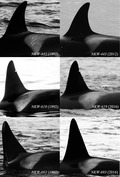

## 1 INTRODUCTION

Life history and other population parameters are key elements in status assessments of animal populations. In particular, mortality rate, population size, and geographic range are the main criteria for evaluation of a species’ extinction risk (IUCN, 2019), because small populations characterized by restricted geographical ranges are less buffered against losses and face an increased risk of extinction (Purvis et al., 2000). Time series of abundance estimates can indicate the extent to which a population may be in decline and estimates of survival and birth rates are important components in the evaluation of conservation measures. Information on abundance is also needed to assess how predators may affect prey populations and how they may respond to fluctuations in prey availability (e.g., Millon et al., 2014; Morissette et al., 2010).

For a wide range of taxa, survival and abundance have been routinely estimated using capture–recapture methods applied to photo‐identification data (e.g., birds: Dugger et al., 2004; felids: Oliver et al., 2011; reptiles: Sreekar et al., 2013; sharks: Gore et al., 2016). In cetacean research, photo‐identification is a noninvasive way to consistently “capture” (first photographic record) and “recapture” (subsequent photographic records) individually recognizable animals over time, using long‐lasting natural markings (Hammond, 1990). This technique offers the possibility to include images from participants other than primary research teams (e.g., citizen science), thus increasing sample size at reduced costs and allowing for data collection in regions for which funding may be limited (Gibson et al., 2020). Best practices in image manipulation, scoring, and cataloguing are important to allow generation of robust capture history datasets for analysis (Urian et al., 2015). Capture–recapture models fitted to capture histories generated from photo‐identification data have been used to obtain estimates of survival rates and abundance for a range of cetacean species (e.g., Arso Civil et al., 2019; Pace et al., 2017; Ramp et al., 2006; Schleimer et al., 2019; Zeh et al., 2002).

Photo‐identification was first applied to killer whales (*Orcinus orca*) in the north‐eastern Pacific in 1973 (Bigg, 1982) and has since led to robust estimates of life‐history parameters for a number of discrete populations worldwide (Durban et al., 2010; Esteban et al., 2016; Fearnbach et al., 2019; Jordaan et al., 2020; Kuningas et al., 2014; Olesiuk et al., 1990, 2005; Pitman et al., 2018; Tixier et al., 2015, 2017). As time series of photo‐identification data have become increasingly available, they have played a central role in identifying population trends and conservation status. For example, a small population size (≤100 individuals), low declining survival rates, and/or low‐to‐no reproductive output were used as basis for management advice on killer whales at Crozet (Guinet et al., 2015; Poncelet et al., 2010; Tixier et al., 2015, 2017), Gibraltar (Esteban et al., 2016), Prince William Sound, Alaska (AT1 group, Matkin et al., 2008), and for the Southern resident population in British Columbia, Canada (COSEWIC, 2008). These studies provided an understanding of underlying threats to long‐term survival of killer whales and also emphasized the need to account for intrapopulation heterogeneity in behavior when assessing demographic trajectories in this species, otherwise risking false trends being detected (see Esteban et al., 2016; Tixier et al., 2015, 2017).

In Norway, photo‐identification studies of killer whales were initiated in the 1980s (Lyrholm, 1988). In this part of the world, killer whales have long been known to mainly feed on Atlantic herring (*Clupea harengus*) and, more specifically, to follow seasonal movements of the Norwegian Spring Spawning stock (hereafter referred to as the NSS herring; Christensen, 1982, 1988; Jonsgård & Lyshoel, 1970; Similä et al., 1996). The NSS herring has gone through major changes in abundance and distribution over the past decades, with recruitment of abundant year classes to the spawning stock often resulting in changes in wintering locations (Dragesund et al., 1997; Huse et al., 2010). Throughout the 1990s, the NSS herring (and killer whales) consistently wintered in the fjord system of Tysfjord‐Vestfjord, where they were readily accessible for study, and killer whales were photo‐identified annually from 1986 through 2003 (Bisther & Vongraven, 1995; Kuningas et al., 2014; Similä et al., 1996). From these 18 years of data, population size, survival, and reproductive rates were estimated for the first time for killer whales in Norway, which were comparable to other apparently healthy killer whale populations (Kuningas et al., 2014). From 2002 onward, as the inshore winter distribution of NSS herring progressively shifted to a new area further offshore (Holst et al., 2004; Huse et al., 2010), lower numbers of killer whales entered the fjords each year until 2008, after which data collection was interrupted for a few years. After the NSS herring started wintering in coastal fjords of Vesterålen and Troms, annual winter photo‐identification surveys were resumed from 2013 (see Jourdain & Vongraven, 2017).

Other major ecological changes occurred in the Norwegian Sea over the past two decades and may have impacted killer whales. The north‐eastern Atlantic mackerel (*Scomber scombrus*) increased in biomass (from ~2 Mt in 2007 to 9 Mt in 2014) and expanded its geographic range north‐ and westward (ICES, 2013; Nøttestad, Utne, et al., 2015). The NSS herring declined from ~12 Mt in 2009 to 5 Mt in 2014 and changed feeding and wintering distributions (ICES, 2013, 2018). A number of cetacean predators of herring (e.g., pilot whales *Globicephala melas* and humpback whales *Megaptera novaeangliae*) seem to have increased in occurrence in the Norwegian Sea (Leonard & Øien, 2020b; Nøttestad, Krafft, et al., 2015), while other abundant baleen whales (e.g., common minke whales *Balaenoptera acutorostrata acutorostrata* and fin whales *Balaenoptera physalus*) may have switched from mainly feeding on planktonic prey to pelagic fish such as herring (see Nøttestad, Krafft, et al., 2015; Nøttestad, Sivle, Krafft, Langård, et al., 2014), implying possible variations in resource competition (see Jourdain & Vongraven, 2017). Recent studies in seasons and locations not previously investigated have documented new prey types, that is, Atlantic salmon (*Salmo salar*; Vester & Hammerschmidt, 2013), Atlantic mackerel (Nøttestad, Sivle, Krafft, Langard, et al., 2014), harbor porpoise (*Phocoena phocoena*; Cosentino, 2015), lumpfish (*Cyclopterus lumpus*; Jourdain et al., 2019), and pinnipeds (Jourdain et al., 2017; Vongraven & Bisther, 2014) for killer whales in Norway, including for individuals known as herring‐eaters (see Jourdain et al., 2019, 2020). These new observations could be the result of enhanced research effort but could also reflect behavioral responses to a changing marine ecosystem. Recent toxicological assessments, which analyzed both fish specialists and individuals who consumed various proportions of fish and pinnipeds, showed that killer whales in Norway carried higher pollution levels than previously assumed, with possible impact on survival and population growth (Andvik et al., 2020). Estimates from line‐transect surveys in the Northeast Atlantic are insufficiently precise to explore whether killer whale abundance in this area may have changed in the last 20 years (2002–2007: 18,821 and 95% CI: 11,525–30,735; 2008–2013: 9563 and 95% CI: 4713–19,403 in Leonard & Øien, 2020b; 2014–2018: 15,056 and 95% CI: 8423–26,914 in Leonard & Øien, 2020a). To investigate how killer whales may have responded to this period of rapid ecosystem change in the Norwegian Sea, new estimates of population parameters are needed.

In this study, we fitted capture–recapture models to a photo‐identification dataset spanning a 32‐year period to generate population parameters for killer whales in northern Norwegian waters. The objectives were (1) to estimate survival rates for the period 1988–2019, including investigating any difference between time periods (i.e., 1988–2008 and 2012–2019) and possible underlying factors and (2) to estimate the size of the population at recent herring wintering grounds in 2012–2019 for comparison with estimates published for the period 1986–2003 (Kuningas et al., 2014). The overall aim was to improve understanding of how killer whales may respond to shifting prey populations in rapidly changing Arctic marine ecosystems.

## 2 MATERIAL AND METHODS

### 2.1 Study area and data collection

**FIGURE 1:**
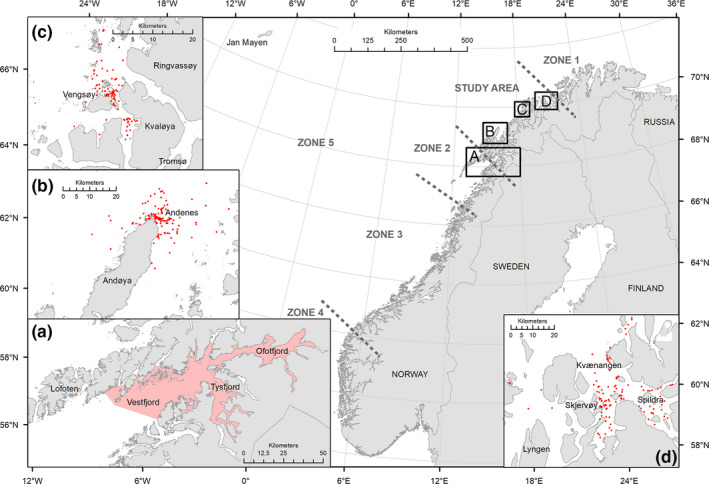
Map showing the study areas where photo‐identification data were collected in northern Norway in 1988–2019: the red‐shaded area in region A indicates where killer whale encounters occurred in 1988–2008 and red plots in regions B (2013–2018), C (2015–2016) and D (2017–2019) indicate exact location at start of killer whale encounters. Killer whale photographs provided by citizen‐science originated from areas B, C, and D in 2012–2019 (not plotted). Adjacent coastal (zones 1–4) and offshore (zone 5) regions from which opportunistic photo‐identifications were available are also shown

Annual photo‐identification surveys were conducted independently by three teams of investigators in Tysfjord‐Ofotfjord‐Vestfjord, in Lofoten (68°19′34.52″N, 15°56′44.38″E) from 1988 to 2008; off Andøya, in Vesterålen (69°16′29.47″N, 16°25′2.29″E) from 2013 to 2018; off Vengsøya‐Kvaløya, in Troms (69°48′52.06″N, 18°38′54.31″E) from 2015 to 2016; and in Kvænangen (70°4′36.22″N, 21°11′29.13″E) from 2017 to 2019, resulting in a study area of ~900 km^2^ for the entire study period (Table 1; Figure 1). During 1988–2008 (referred to as Period 1), fieldwork took place between October and January. Data collection shifted to November–February in 2012–2019 (referred to as Period 2) in response to the later arrival of herring and killer whales in the fjords. Hereafter, each field season is referred to by the initial year (e.g., winter 2012–2013 is designated as 2012).

**TABLE 1:** Total numbers of observation days, killer whale encounters (when known), photographs (total collected regardless of quality), individual killer whales identified (IDs), and of fair‐to‐excellent quality (see Section 2) identifications including resightings, as contributed by the three research teams (DV/AB: Dag Vongraven and Anna Bisther, TS/SK: Tiu Similä and Sanna Kuningas, NOS: Norwegian Orca Survey) and citizen‐science (CS) in 1988–2019 in the study area, and which contributed to building the capture histories of the 1236 killer whales included in this study

Source	Years	Days	Encounters	Photographs	IDs	Identifications
TS/SK	1988–2008	272	318	12,420	316	2415
DV/AB	1990–1996	103	N/A	N/A	179	452
NOS	2013–2019	151	422	94,900	1032	1843
CS	2012–2019	303	N/A	66,900	694	1345

Note

A number of observation days overlapped between DV/AB and TS/SK because the two research teams operated independently but at the same time of year and within the same region.

**FIGURE 2:**
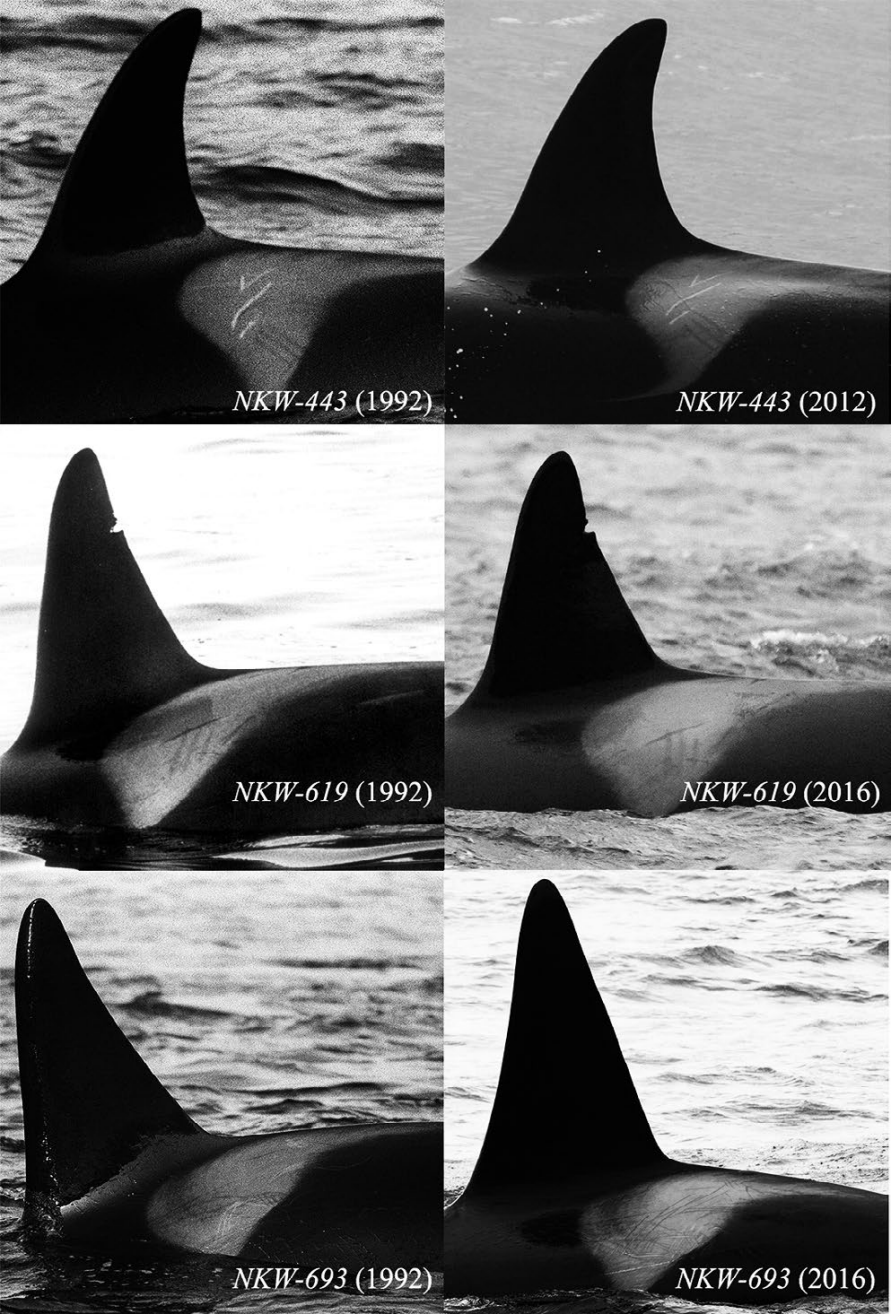
Sample of identification photographs showing the persistence of scarring and pigmentation patterns of the saddle patch and nicks in the dorsal fin and thus their reliability for long‐term re‐identification of individual killer whales in Norway. Distinctively taller dorsal fin for NKW‐693, compared to NKW‐443 and NKW‐619 for which dorsal fin did not develop over the course of the study, further illustrates how sex could be readily determined based on morphological features for most identified individuals

In both periods, surveys were carried out opportunistically or using sighting reports obtained from other vessels in the area, with the aim of maximizing the number of killer whales found. A similar approach was maintained in 2013–2014 when whale‐watching rigid inflatable boats were used as research platforms. When a group (defined as individuals in apparent association and acting in a coordinated manner during the observation period) was encountered, left‐sided identification photographs were taken following the protocols described by Bigg (1982; Figure 2). Efforts focused on photographing as many individuals as possible in each encountered group (hereafter referred to as an encounter), regardless of individuals’ size, behavior, or distinctiveness to minimize heterogeneity of capture probabilities (Hammond, 2010). When all or most individuals in the encounter were believed to have been photographed, the research vessel left the animals and resumed its search for other killer whale groups. Photographs were taken with SLR cameras and Kodak T MAX or Ilford HP5 400 ASA films in 1988–2000 and DSLR cameras in 2001–2019, all equipped with 200‐ or 300‐mm lenses. Supplementary photographs, with reliable information on date and time, collected from wildlife photographers and members of the general public within the study area were also used for identification purposes in 2012–2019 (Table 1; Figure 1).

### 2.2 Photo‐identification

#### 2.2.1 Photograph processing

Processing photographs required the films to be inspected using a stereoscopic microscope until 2000, after which digital images were viewed and enhanced in Adobe Photoshop. The three teams of investigators followed similar photo‐identification protocols. For each encounter, individuals were identified from left‐sided photographs using nicks, shape, and size of the dorsal fin, alongside scarring and pigmentation patterns of the adjacent gray saddle patch as per Bigg (1982; Figure 2). The best photograph of each individual from each encounter was selected and rated for (1) quality (poor, fair, good, excellent) based on combined criteria of sharpness, contrast, and size of the dorsal fin relative to the frame; (2) angle of the killer whale relative to the photographer (parallel, slight angle, angle); and (3) proportion of the saddle patch visible (top 1/3, top 2/3, or fully visible). Each identified individual was matched against an existing catalogue of previously identified individuals (see published catalogue 2007–2021: Jourdain & Karoliussen, 2021). If a match was found, the individual received a new record of where and when it was seen together with corresponding photograph scoring. If no match was found, the previously unidentified individual was assigned a unique identification (ID) number, added to the ID catalogue and received its first sighting record. A database listing individuals’ sighting histories was held independently by each research team (Table 1). For each encounter, unidentifiable individuals lacking features for reliable long‐term identification were differentiated from each other using temporary subtle skin markings (e.g., body scars, lesions) and pigmentation of the eye patch from photographs of fair‐to‐excellent quality. This information on the number of identified and unidentified individuals in an encounter was used to estimate the proportion of identifiable individuals in the population and correct capture–recapture estimates of abundance (see below).

#### 2.2.2 Comparing ID catalogues

To build a database common to all three studies, images of individual killer whales were systematically cross‐matched across all three ID catalogues (Table 1). Potential matches were evaluated by five of the authors (EJ, TG, TS, SK, DV) and by an external analyst with >40 years of experience with photo‐identifying killer whales (Graeme Ellis). A match was considered certain only when accepted by all. Individuals found in more than one catalogue were renamed to a unique ID number and their sighting histories, as logged independently by the different investigators, were combined.

### 2.3 Characterization of individuals

#### 2.3.1 Determining sex and age class

Using clear morphological evidence of physical maturity, adult males were identified based on a distinctively taller dorsal fin (Bigg, 1982; Olesiuk et al., 1990; Figure 2). Other individuals of apparent mature size, seen in close and consistent association with a calf (in echelon position) on at least two encounter days, or showing no development of the dorsal fin in at least 3 years, were categorized as adult females (Figure 2). Individuals for which sex could not be determined were categorized as “unknowns.” These individuals could be either subadult males or females, or adult females.

#### 2.3.2 Assessing ranging patterns

To assess how the study area compared to ranging capacities of killer whales identified from annual winter surveys, we compiled additional photographic records collected from citizen‐science in adjacent coastal and offshore regions for these individuals (Figure 1).

### 2.4 Mark–recapture analyses

#### 2.4.1 Data selection

To be considered marked (re‐identifiable) and to be retained for analysis, an individual had to have a minimum of one primary feature, defined as (a) at least three scars on the saddle patch; or (b) at least two nicks in the dorsal fin, or a minimum of two secondary features. Secondary features were defined as (i) one or two scars on the saddle patch, (ii) a single nick in the dorsal fin, and (iii) distinctive pigmentation of the saddle patch (Figure S1). In addition, only identifications from photographs of fair‐to‐excellent quality of killer whales describing a parallel or only slight angle relative to the photographer, and for which the full dorsal fin and at least the top 2/3 of the saddle patch were visible were retained for analysis (Figure S1). Individuals (including calves) lacking permanent markings were excluded from all analyses but were used to estimate the proportion of identifiable individuals in the population (see below).

#### 2.4.2 Cormack–Jolly–Seber (CJS) models

To estimate annual survival probabilities using CJS models (Lebreton et al., 1992), capture histories were built by pooling sightings recorded during the same annual winter season and by treating each year as a sampling occasion. Prior to running models, we ran goodness‐of‐fit (GOF) tests implemented in the R (version 4.0.2; R Core Team, 2018) package R2ucare (Gimenez et al., 2018) to test for lack of fit of the global CJS model. Through specialized interpretable test components, this approach can identify features of the data that underlie departure from model assumptions. In particular, component Test 3.SR tests for equal probability of recapture between newly and previously captured individuals (Pradel et al., 1997), and Test 2.CT tests for equal recapture probability between individuals encountered and not encountered in a given sampling occasion (Pradel, 1993). These tests can identify features of the data that are typically caused by a transience effect, resulting from the presence of transient individuals (defined as having been seen only once), and trap‐dependence, in which recapture probability is influenced by whether or not an individual was captured during the previous sampling session, respectively. However, these features could also be the result of other features of the data. The global test, combining all test components, was used to assess the general goodness of fit of the CJS model.

CJS models were fitted to annual sex‐specific capture histories for 1988–2019 (thus excluding unknown sex animals) to estimate adult apparent survival probability (*φ*; incorporating any permanent emigration) between years and recapture probability (*p*) for each year. Gap years (2009, 2010, 2011 with no data available) were included in the full time series by fixing recapture probability to 0 for these years. A set of candidate models was constructed in which apparent survival and recapture probabilities were (using conventional notation): constant over time (.), varied annually (*t*), or displayed a linear temporal trend (*T*) (Lebreton et al., 1992). In addition to incorporating a temporal trend, we explored the effect of modeling Period 1 and Period 2 as distinct time periods (*period*). A sex‐effect (*s*) on both survival and recapture probabilities was also tested. GOF Test 2.CT indicated a behavioral (“trap”) response (see Section 3) and justified testing the effect of trap‐dependence (*td*) when modeling recapture probabilities. Trap‐dependence was implemented using an individual time‐varying covariate comprising dummy variables (0 and 1) depending on whether or not an individual was seen on the previous occasion. Lack of fit in GOF Test 3.SR (see Section 3) justified testing the effect of transience on estimates of apparent survival. This was achieved by building time‐since‐marking models with two classes (*trans*), in which survival probability was estimated for the first annual interval after first capture (first class) and also for all subsequent annual intervals (second class). Additive (+) and interactive (*) models were constructed to test for combinations of effects on *φ* and *p*. Overdispersion in the data was evaluated by calculating the variance inflation factor (*ĉ*, “c‐hat”) as global GOF test *X* ^2^/degrees of freedom (Lebreton et al., 1992).

The probability of apparent survival is the product of surviving from one sampling occasion to the next and of returning to the study area. We investigated whether differences in residency patterns could influence estimates of apparent survival in Period 2 (2012–2019). Residency groups were identified by categorizing individuals following methods described by Schleimer et al. (2019).

Sighting histories of all individuals (males, females, unknowns) in 2012–2019 were used to calculate individuals’ yearly (YSR) and seasonal (SSR) sighting rates with: YSR = number of years in which seen/total number of years since first identification, and SSR = number of days in which seen/total number of days since first identification in a given season. Individuals first identified in 2018 and 2019 were excluded due to insufficient years with sighting data to reliably evaluate residency patterns. Agglomerative Hierarchical Clustering (AHC) was conducted using the *hclust* function in R to classify individuals based on similar sighting rates. To allow for direct comparison of the two rates, YSR and SSR were standardized (relative to the median and the median absolute deviation) beforehand using the *scale* function in R. In the AHC, Euclidean distance was chosen as a measure of dissimilarity and to compute proximity matrices between individuals using Ward's method. This clustering method merges the closest individuals (data points) into clusters based on a proximity matrix. The most appropriate number of residency groups was chosen based on obvious main clusters identified visually in the resulting dendrogram.

CJS models were fitted separately to the residency groups identified by the AHC analysis. For each group, both survival and recapture probabilities were allowed to be constant over time (.), vary annually (*t*), or display a linear temporal trend (*T*).

#### 2.4.3 Robust design models

Robust design (RD) models were fitted to the capture histories of all individuals (males, females, unknowns) in Period 2 to estimate the annual number of killer whales using the study area and to evaluate the extent of temporary emigration from the study area between years (Kendall et al., 1997). Each annual winter season (year) was considered as a primary sampling occasion. Each survey area was covered in every week in all years, so weeks within each winter season were treated as secondary sampling occasions (Table 2). Candidate models were built to incorporate effects that were constant over time (.), varied over time (*t*), had a linear temporal trend (*T*), and/or a transience effect (*trans*) on survival probabilities (φ; GOF Test 3.SR was marginally significant—see Section 3). Capture and recapture probabilities were assumed equal in all models (*p* = *c*) and were modeled to vary by primary sampling occasion alone (*session*) or by both primary and secondary sampling occasion (session*time). The probability of temporary emigration from the study area between years (primary occasions) was modeled using the parameters γ′ (probability of being outside the study area conditional on being outside the study area in the previous year) and γ″ (probability of being outside the study area conditional on being inside the study area in the previous year). γ″ can thus be interpreted as the annual probability of temporary emigration and 1 – γ′ as the annual probability of re‐immigration. Temporary emigration was modeled as: random (γ′ = γ″), Markovian (γ′ ≠ γ″), or no emigration (γ′ = 1; γ″ = 0). Temporary emigration parameters were modeled as either constant (.) or varying over time (*t*).

**TABLE 2:** Total number of captures in each secondary occasion (weeks within field seasons) that made up each primary period (years) in the dataset 2012–2019 used for fitting robust design models to the capture histories of all individuals (i.e., males, females, unknowns)

	1	2	3	4	5	6	7	8	9	10	11	12	13	14	15	16	17
2012	18	20															
2013	11	13	21	22	45												
2014	8	16	11	6	16	28	37	38									
2015	18	17	45	99	78	22	31	13	31	35	121	44	40	24	37	17	16
2016	29	108	133	123	33	8	38	32	22	27							
2017	48	61	82	104	13	14	11	19									
2018	50	47	28	111	121	47	25	13	18	22	28						
2019	77	155	85	61	33	16	25										

#### 2.4.4 POPAN models

To obtain an alternative estimate of the size of the “super‐population,” defined as the total number of killer whales that was in the study area at some point in time, the POPAN parameterization of the Jolly–Seber model (Schwarz & Arnason, 1996) was fitted to the capture histories of all individuals (males, females, unknowns) in Period 1 (1988–2008) and in Period 2 (2012–2019). Other parameters in the POPAN model are the probability of apparent survival (*φ*), capture (*p*), and recruitment into the study area from the super‐population (*pent*). All these parameters were modeled as constant (.), varying over time (*t*), or as a trend over time (*T*). Because GOF Test 3.SR was significant (marginally for Period 2—see Section 3), we also built time‐since‐marking models with two classes (*trans*) to account for transience effects in survival probabilities, with additive and interactive effects with (*t*) and (*T*). Estimates of annual population size were also derived from the models.

#### 2.4.5 Model selection, adjustment for overdispersion, and model‐averaging

The support that candidate CJS and POPAN models received from the data was assessed using quasi‐likelihood AIC for small sample size (QAICc), obtained by adjusting AICc for overdispersion using estimated *ĉ* (except for the high residency group which did not show overdispersion—see Section 3). Estimates of parameters of interest were obtained by model‐averaging over models within delta‐QAICc ≤10 of the lowest QAICc, considered to receive some support from the data (Burnham & Anderson, 2002). GOF tests are unavailable for RD models so neither overall model fit nor overdispersion in the data could be assessed. Therefore, AIC_C_ was used to assess relative model fit.

All capture–recapture analyses were conducted using the package RMark v.2.2.7. (Laake & Rexstad, 2008) in R.

#### 2.4.6 Proportion of identifiable individuals

The proportion of identifiable individuals in the population in each year, *θ*, was estimated by fitting a binomial generalized linear model with logit link function to the number of identified and unidentified individuals in encountered groups, where this could be determined. Total population size was estimated as:

$${\hat{N}}_{\text{total}}=\frac{\hat{N}}{\hat{\theta }}$$

where $\hat{N}$ is the capture–recapture estimate of the number of identifiable animals, with coefficient of variation (CV) estimated using the delta method as:

$${\text{CV}}_{N_{\text{total}}}^{2}={\text{CV}}_{N}^{2}+{\text{CV}}_{\theta }^{2}$$

and 95% confidence intervals calculated assuming a log normal distribution as *N*_total_/c to c*N_total_, where:

$$c=e^{1.96\sqrt{\ln(1+CV_{N_{\text{total}}}^{2})}}.$$

## 3 RESULTS

### 3.1 Data summary

**FIGURE 3:**
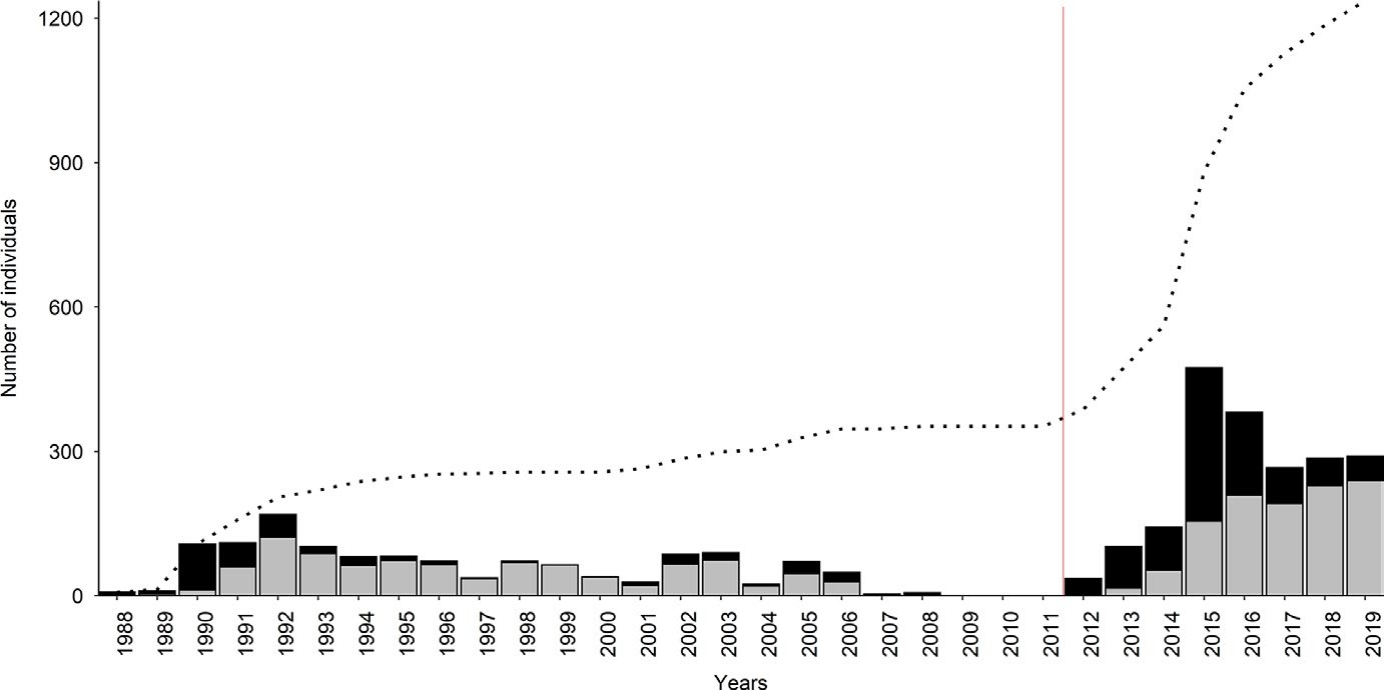
Number of killer whales identified for the first time (black) and previously identified (gray) in each year (bar plots) and the cumulative discovery curve of new individuals in 1988–2019. The red solid line indicates the transition to Period 2 (2012–2019)

**FIGURE 4:**
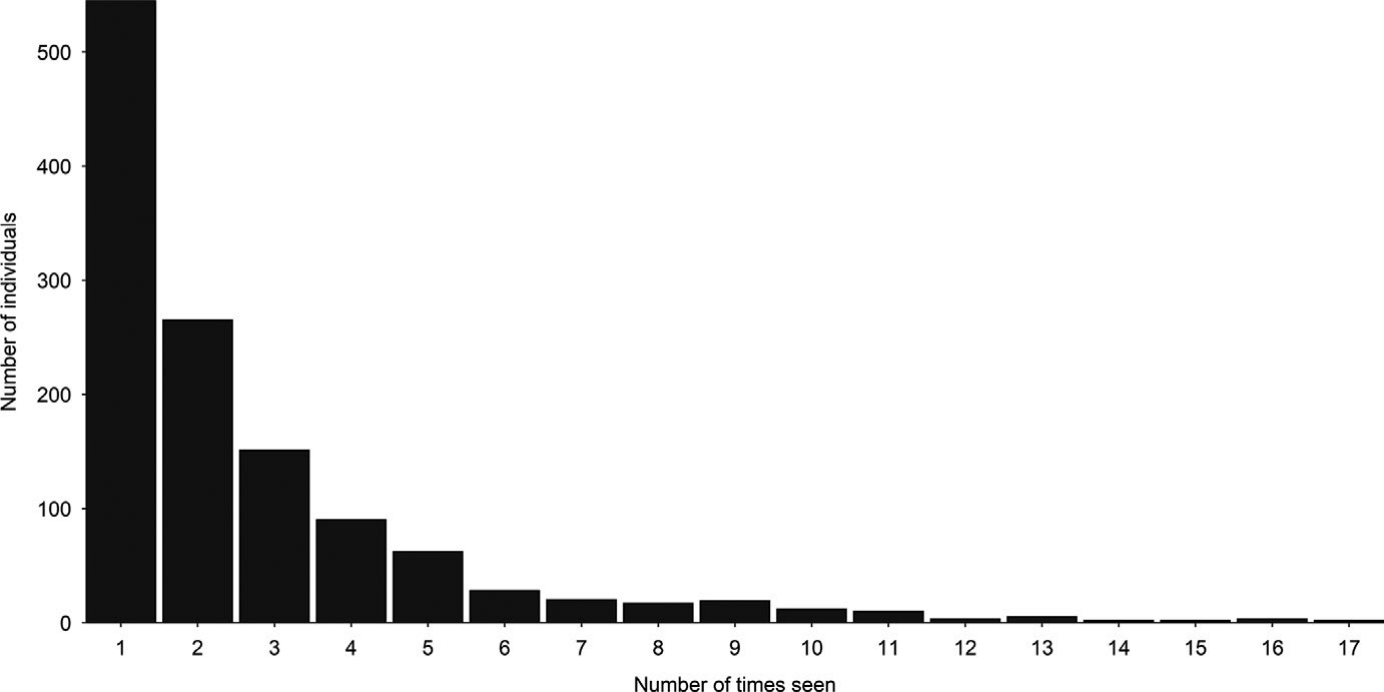
Frequency of capture (number of years in which seen) for the 1236 individual killer whales identified in 1988–2019

**FIGURE 5:**
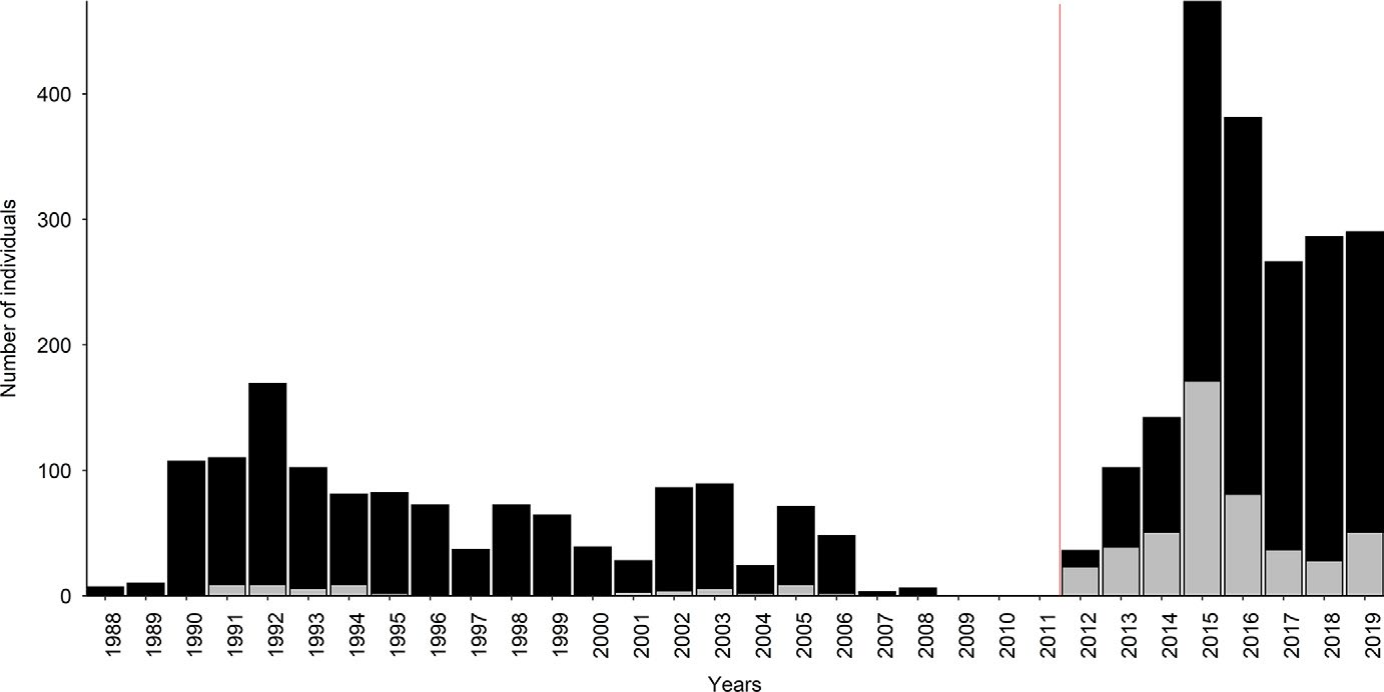
Number of individuals seen in only 1 year (transients, gray) relative to the total number of identified individuals (black and gray combined) in each year. The red solid line indicates the transition to Period 2 (2012–2019)

A total of 672 observation days (unique dates) in 1988–2019 resulted in 6055 identifications and 3284 annual captures of 1236 individual killer whales throughout the study period (Table 1; Figures 1, 2, 3, S2). Comparing the three ID catalogues (see Table 1), 179 matches were identified between the catalogues held by TS/SK and DV/AB, 72 matches between Norwegian Orca Survey (NOS) and DV/AB and 151 matches between NOS and TS/SK, including 148 individuals seen in both periods (Table S3; Figures 2 and 3). Overall, 691 (56%) individuals were seen in two or more years (Table S3; Figure 4). While the rate of new identifications leveled off toward the end of Period 1, it increased again after fieldwork was resumed at newly established herring wintering grounds from 2012 (Table 1; Figures 1 and 3). The proportion of transients (animals seen only once) relative to the total number of identified individuals in each year peaked in 2015 and 2016 and averaged 30% (SD = 18%) in Period 2, compared to 5% (SD = 5%) in Period 1 (Figure 5). Sex was reliably determined for 960 of the 1236 individuals, including 719 males and 241 females, while 276 were categorized as unknowns (Table S4). Such a disproportionate sex ratio in the ID catalogue may be explained by adult males being more identifiable due to their tendency to bear more markings than adult females and subadults; only 6% of the unmarked individuals in 2015–2019 were adult males. In 1988–2019, 71 (6%) killer whales identified at herring wintering grounds had also been photo‐identified in at least one adjacent region, in one or multiple years (Table 3; Figure 1).

**TABLE 3:** Number of photo‐identified killer whales that were opportunistically photo‐identified in adjacent coastal (zones 1–4) and offshore (zone 5) regions in addition to their winter records in the study area. Zones (Z) match region subdivision from Figure 1

	Z1	Z2	Z3	Z4	Z5	Any zone	M/F/U	High/Low
Period 1	0	0	26	0	0	26	12/13/1	–
Period 2	3	28	11	0	7	45	26/17/2	18/22
Full series	3	28	37	0	7	71	39/30/2	–

Note

The sex ratio (males/females/unknowns) and the number of individuals assigned to each of the high and low residency groups (Period 2) are also shown. Some individuals may have been seen in multiple zones meaning that “Any Zone” is not the sum of the Z‐columns

### 3.2 Apparent survival rates 1988–2019

**FIGURE 6:**
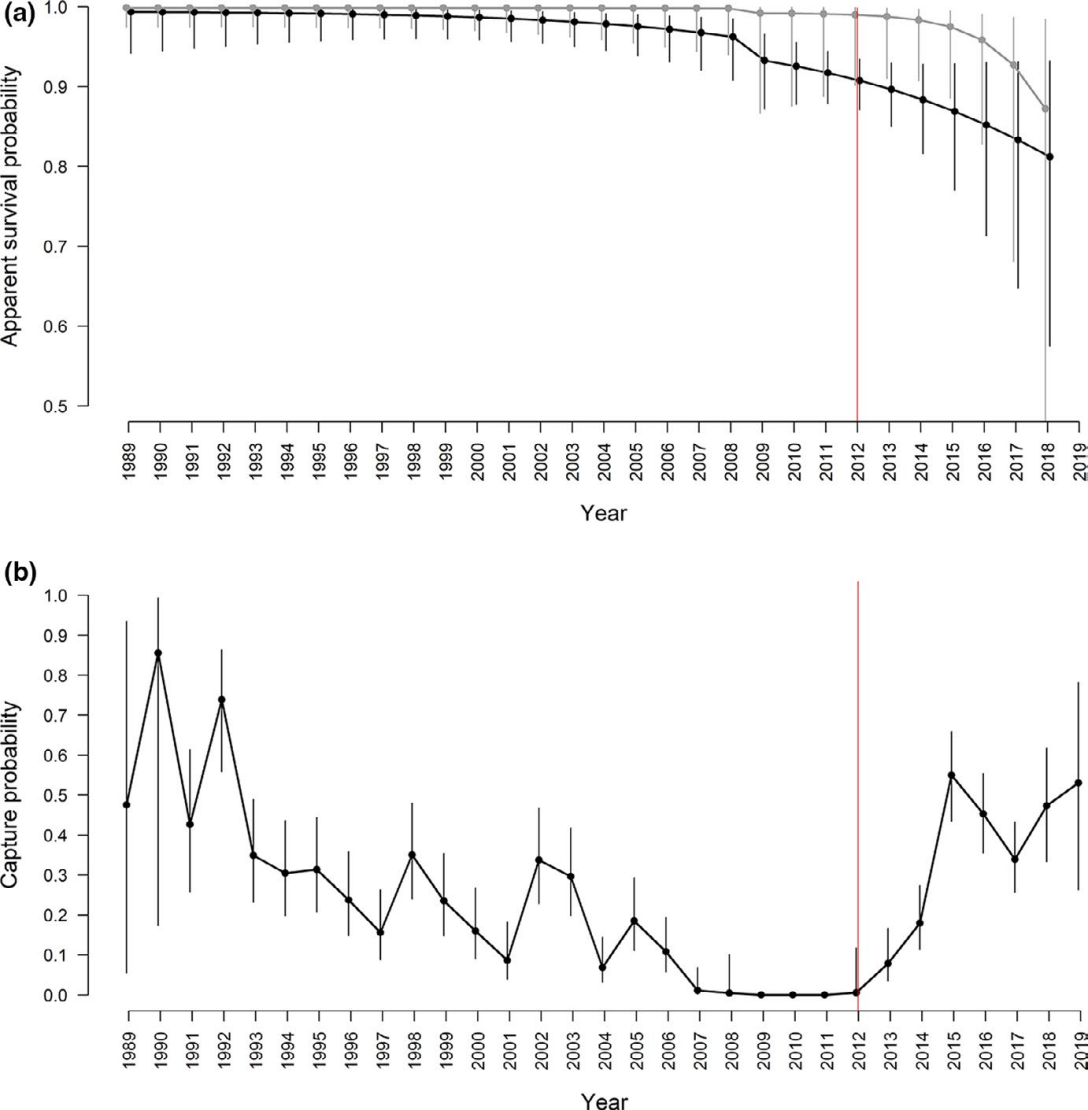
Model‐averaged probabilities of (a) apparent survival and (b) recapture with 95% CI for adult male (black) and female (gray) killer whales in 1988–2019, as estimated from the four best‐supported CJS models listed in Table 5. The red solid line indicates the transition to Period 2 (2012–2019)

GOF tests 2.CT and 3.SR indicated a lack of fit of the CJS model (Table 4), which was addressed by fitting models that included the effects of trap‐dependence on recapture probability and transience on apparent survival probability. CJS models accounting for combined (additive or interactive) effects of sex, transience, and blocks of time or linear temporal trend on survival carried all the QAICc weight (Table 5). Model‐averaged estimates of apparent survival declined from 0.998 (± 0.002 SE) for females and 0.985 (± 0.009 SE) for males in Period 1 to 0.955 (± 0.027 SE) and 0.864 (± 0.038 SE), respectively, in Period 2 (values averaged across years for each sex and in each period; Figure 6a). Preliminary modeling of the two periods separately indicated that the data gap between periods did not influence estimates of survival in the two periods when modeling the entire dataset. Average apparent survival was lower for transients (geometric mean Period 1: 0.907 ± 0.043 SE, Period 2: 0.697 ± 0.070 SE). The models for recapture probabilities that accounted for additive effects of time and trap‐dependence received all the QAICc weight (Table 5). Model‐averaged recapture probabilities varied considerably throughout the study period, reaching maxima (>0.53) at the beginning and toward the end of the time series (Figure 6b).

**TABLE 4:** Results of the four directional goodness‐of‐fit tests (GOF), the global combined test of overall CJS model fit, and the variance inflation factor (ĉ, “c‐hat”) calculated as *X* ^2^/degrees of freedom

Dataset	3.SR	3.SM	2.CT	2.CL	Global test	ĉ
*1988–2019*						
MF	*X* ^2^ = 76.903 df = 23 *p*< .001	*X* ^2^ = 31.840 df = 33 *p* = .525	*X* ^2^ = 140.885 df = 26 *p*< .001	*X* ^2^ = 75.809 df = 41 *p* = .001	*X* ^2^ = 325.437 df = 123 *p*< .001	2.64
*Period 1*						
MFU	*X* ^2^ = 80.808 df = 15 *p*< .001	*X* ^2^ = 18.045 df = 18 *p* = .453	*X* ^2^ = 71.112 df = 17 *p*< .001	*X* ^2^ = 53.407 df = 33 *p* = .014	*X* ^2^ = 223.372 df = 83 *p*< .001	2.69
*Period 2*						
MFU	*X* ^2^ = 20.273 df = 6 *p* = .002	*X* ^2^ = 10.207 df = 6 *p* = .116	*X* ^2^ = 7.190 df = 5 *p* = .207	*X* ^2^ = 4.025 df = 4 *p* = .403	*X* ^2^ =41.695 df = 21 *p* = .005	1.98
HIGH	*X* ^2^ = 0 df = 1 *p* = 1	*X* ^2^ = 5.322 df = 4 *p* = .256	*X* ^2^ = 3.434 df = 4 *p* = .488	*X* ^2^ = 1.323 df = 3 *p* = .724	*X* ^2^ =10.079 df = 12 *p* = .609	0.84
LOW	*X* ^2^ = 7.926 df = 5 *p* = .160	*X* ^2^ = 10.383 df = 6 *p* = .109	*X* ^2^ = 7.110 df = 5 *p* = .213	*X* ^2^ = 10.778 df = 4 *p* = .029	*X* ^2^ = 36.197 df = 20 *p* = .015	1.81

Note

Datasets are MF: sex‐specific, MFU: males, females, unknowns, HIGH: high residency group, LOW: low residency group.

**TABLE 5:** Summary of the best‐supported candidate CJS models (≤10 ΔQAICc) for 1988–2019 used for model‐averaging the probability of apparent survival (*φ*) and of recapture (*p*) accounting for additive (+) or interactive (*) effects of time (*t*), a linear temporal trend (*T*), sex (*s*), periods of time (*period*), transience (*trans*), and/or trap‐dependence (*td*)

Model	QAICc	ΔQAICc	QAIC weight	Q deviance	Number of parameters
φ(s*T*trans) p(t + td)	2666.064	0	0.472	2584.732	40
φ(s*period*trans) p(t + td)	2667.297	1.233	0.255	2585.964	40
*φ(s + period + trans) p(t + td)*	2667.573	1.509	0.222	2594.493	36
*φ(s + T + trans) p(t + td)*	2670.499	4.434	0.051	2597.418	36

### 3.3 Residency groups and survival rates 2012–2019

**FIGURE 7:**
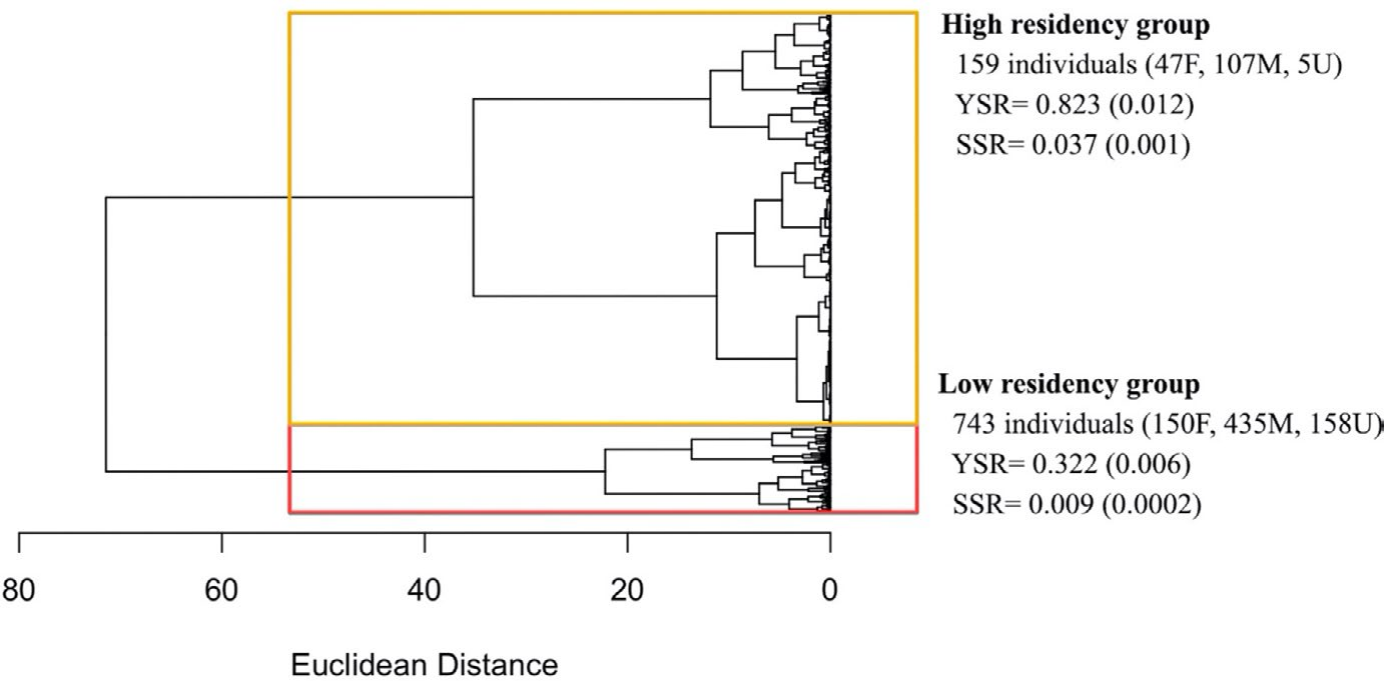
Dendrogram showing the results of the Agglomerative Hierarchical Cluster (AHC) analysis conducted on individuals seen in 2012–2019 on the basis of dissimilarity in yearly (YSR) and seasonal sighting rates (SSR). Sex ratio (females/males/unknowns) for each cluster is also shown

Out of the 902 killer whales encountered in 2012–2019 and first identified before 2018 (see Section 2), the AHC results (Figure 7) indicated two main clusters in which 159 individuals were assigned to a first cluster characterized by high yearly (0.823 ± 0.012 SE) and seasonal (0.037 ± 0.001 SE) sighting rates, hereafter referred to as the “High residency group.” The remaining 743 whales were assigned to a second cluster characterized by low yearly (0.322 ± 0.006) and seasonal (0.009 ± 0.0002 SE) sighting rates, hereafter referred to as the “Low residency group.” A Mann–Whitney Wilcoxon test for non‐normally distributed data confirmed a significant difference in both yearly (*W* = 1850.5, *p* < .001) and seasonal (*W* = 601, *p* < .001) sighting rates between the two residency groups. Figure 7 could be interpreted as showing three clusters, rather than two. We estimated apparent survival rates independently for the three indicated clusters. Results (not shown) indicated that estimated survival was the same for two of the clusters and were thus no more informative that the results for two clusters.

**FIGURE 8:**
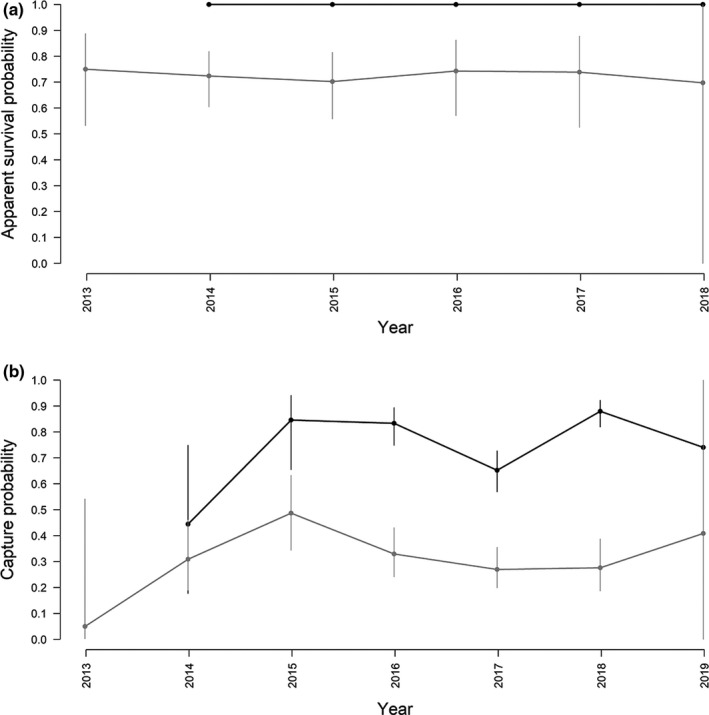
Non‐sex‐specific probabilities of (a) survival and (b) recapture with 95% CI for the High residency group (black) and the Low residency group (gray), as obtained from the best‐supported CJS models listed in Table 6. Note: there were no captures in the High residency group in 2012

No lack of fit of the CJS models was detected from the GOF tests run on each of the two residency groups (Table 4). For the High residency group, the most supported CJS model received 73% of the AICc weight and estimated apparent survival at 1 (95% CI: 0.99–1.00) (Table 6; Figure 8a). This is explained by all 159 killer whales in this residency group still being alive at the end of this short second study period (2012–2019). Model‐averaged estimates of apparent survival were much lower for the whales assigned to the Low residency group (geometric mean: 0.731 ± 0.075 SE; Figure 8a). In this group, individuals also had consistently lower recapture probabilities than the high residency group, confirming reduced fidelity to the area for these whales (Figure 8b).

**TABLE 6:** Most‐supported CJS model for the High residency group and best‐supported candidate models (≤10 ΔQAICc) for the Low residency group used for model‐averaging the probability of apparent survival (*φ*) and of recapture (*p*), both allowed to be constant (.), vary by time (*t*) or display a linear temporal trend (*T*)

Model	AICc/QAICc	ΔAICc/ΔQAICc	AIC/QAIC weight	Deviance	Number of parameters
*High residency group (616 captures)*					
*φ(*.*) p(t)*	616.180	0	0.734	76.375	7
*Low residency group (1193 captures)*					
*φ(.) p(t)*	1329.559	0	0.625	109.296	8
*φ(T) p(t)*	1331.589	2.030	0.226	109.293	9
*φ(t) p(t)*	1333.116	3.557	0.106	100.600	14
*φ(t) p(.)*	1336.539	6.980	0.019	116.276	8
*φ(t) p(T)*	1338.005	8.446	0.009	115.709	9
*φ(.) p(.)*	1338.340	8.781	0.008	130.196	2

### 3.4 Temporary emigration

Robust design models that included random temporary emigration, either constant or time varying, carried most of the AIC weight (74%), although models featuring Markovian temporary emigration also received some support from the data (26% of the AIC weight, Table 7). Models with no temporary emigration had no support. Only models with capture probability varying by both primary and secondary sampling occasions were supported. Model‐averaged survival probabilities were similar to those obtained with the CJS (Figure S5a). Model‐averaged capture probabilities varied considerably within and between primary periods, ranging from 0.013 (± 0.006 SE) to 0.392 (± 0.030 SE; Figure S5b). Average annual probability of emigration and re‐immigration were γ″ = 0.148 (± 0.095 SE) and 1 – γ′ = 0.760 (± 0.215 SE), respectively.

**TABLE 7:** Summary of the best‐supported candidate models (≤10 ΔQAICc) obtained when fitting robust design models to the dataset 2012–2019 and used for model‐averaging the probability of apparent survival (*φ*), the probability of being outside the study area conditional on being outside the study area in the previous year (*γ*′) and the probability of being outside the study area conditional on being inside the study area in the previous year (*γ″*)

Model	AICc	ΔAICc	AIC weight	Deviance	Number of parameters
*φ(t + trans) γ″(t) = γ*′*(t)*	−1371.205	0.000	0.364	5025.278	91
*φ(t + trans) γ″(.) = γ*′*(.)*	−1369.319	1.885	0.142	5039.940	85
φ(t*trans) γ″(t) = γ′*(t)*	−1368.863	2.341	0.113	5014.786	97
*φ(t + trans) γ″(*.*)* ≠ *γ*′*(*.*)*	−1368.590	2.615	0.098	5038.545	86
*φ(t + trans) γ″(t)* ≠ *γ*′*(*.*)*	−1368.502	2.703	0.094	5025.846	92
*φ(trans) γ″(t) = γ*′*(t)*	−1367.015	4.190	0.045	5042.245	85
φ(t*trans) γ″(.) = γ′*(.)*	−1366.219	4.985	0.030	5030.263	91
φ(t*trans) γ″(.*)* ≠ *γ*′*(*.*)*	−1365.801	5.404	0.024	5028.547	92
*φ(t) γ″(.) = γ*′*(.)*	−1365.063	6.142	0.017	5046.321	84
*φ(T + trans) γ″(t)* = *γ*′*(t)*	−1364.977	6.227	0.016	5042.157	86
*φ(t) γ″(*.*)* ≠ *γ*′*(*.*)*	−1364.780	6.424	0.015	5044.480	85
φ(t*trans) γ″(t) ≠ *γ*′*(*.*)*	−1364.531	6.674	0.013	5016.975	98
φ(T*trans) γ″(t) = γ′*(t)*	−1362.946	8.258	0.006	5042.061	87
*φ(t + trans) γ″(*.*)* ≠ *γ*′*(t)*	−1362.856	8.348	0.006	5031.491	92
*φ(t) γ″(t) = γ*′*(t)*	−1362.848	8.356	0.006	5035.768	90
*φ(t) γ″(t)* ≠ *γ*′*(*.*)*	−1362.400	8.805	0.004	5034.083	91
*φ(T + trans) γ″(t)* ≠ *γ*′*(*.*)*	−1361.586	9.619	0.003	5043.421	87

Note

For apparent survival, single, additive (+) or interactive (*) effects were modeled as constant (.), time‐specific (*t*), with a linear temporal trend (*T*) and accounting for transience (*trans*). All listed supported models had capture probabilities varying by primary and secondary sampling occasion.

### 3.5 Population size

When fitted to data from Period 1, POPAN models that accounted for a temporal trend (*T*) in recruitment from the super‐population into the study area (*pent*) received >87% of the QAICc weight (Table 8). Models with *pent*(*t*) were unable to estimate all parameters and were therefore excluded from consideration. When fitted to data from Period 2, POPAN models that included a temporal trend (*T*) or a time effect (*t*) on *pent* received equal support from the data (48% and 51% of the QAICc weight, respectively), while models with constant *pent* carried low weight (<2% of the QAICc weight; Table 9).

**TABLE 8:** Summary of the best‐supported candidate models (≤10 ΔQAICc) obtained when fitting POPAN models to Period 1 (1988–2008) and used for model‐averaging the probability of apparent survival (*φ*), of recapture (*p*) and of recruitment into the study area from the super‐population (*pent*), accounting for single, additive (+) or interactive (*) effects of time (*t*), a linear temporal trend (*T*), and transience (*trans*) or set constant (.)

Model	QAICc	ΔQAICc	QAICc weight	*Q* deviance	Number of parameters
*φ (.) p(t) pent(T)*	1508.323	0	0.403	158.738	25
*φ (T) p(t) pent(T)*	1509.484	1.161	0.226	157.817	26
*φ (trans) p(t) pent(T)*	1510.157	1.834	0.161	158.490	26
*φ (T+trans) p(t) pent(T)*	1511.415	3.092	0.086	157.662	27
*φ (.) p(t) pent(.)*	1512.243	3.920	0.057	164.736	24
*φ (T) p(t) pent(.)*	1513.402	5.079	0.032	163.817	25
*φ (trans) p(t) pent(.)*	1514.056	5.733	0.023	164.471	25
*φ (T+trans) p(t) pent(.)*	1515.32	6.997	0.012	163.653	26

**TABLE 9:** Summary of the best‐supported candidate models (≤10 ΔQAICc) obtained when fitting POPAN models to Period 2 (2012–2019) and used for model‐averaging the probability of apparent survival (*φ*), of recapture (*p*) and of recruitment into the study area from the super‐population (*pent*), accounting for single, additive (+) or interactive (*) effects of time (*t*), a linear temporal trend (*T*) and transience (*trans*) or set constant (.)

Model	QAICc	ΔQAICc	QAICc weight	*Q* deviance	Number of parameters
*φ(t) p(t) pent(T)*	1922.582	0	0.264	−1744.355	24
*φ(.) p(t) pent(t)*	1923.441	0.859	0.172	−1741.459	23
*φ(.) p(t) pent(T)*	1924.060	1.479	0.126	−1730.686	18
*φ(t) p(t) pent(t)*	1924.799	2.217	0.087	−1752.353	19
*φ(T) p(t) pent(t)*	1924.862	2.280	0.084	−1742.075	18
*φ(trans) p(t) pent(t)*	1925.144	2.562	0.073	−1741.793	18
*φ(T) p(t) pent(T)*	1925.900	3.319	0.050	−1730.872	17
*φ(trans) p(t) pent(T)*	1925.961	3.380	0.049	−1730.811	17
*φ(t + trans) p(t) pent(t)*	1926.848	4.267	0.031	−1752.354	17
*φ(T + trans) p(t) pent(t)*	1926.851	4.269	0.031	−1742.124	14
*φ(T + trans) p(t) pent(T)*	1927.901	5.319	0.018	−1730.900	13
*φ(t) p(t) pent(.)*	1929.439	6.857	0.009	−1735.461	13
*φ(t) p(T) pent(t)*	1930.719	8.137	0.005	−1734.181	12
*φ(.) p(t) pent(.)*	1932.375	9.793	0.002	−1720.347	11

In Period 1, the proportion of identifiable individuals in the population was 0.556 (± 0.052 SE) for 1990–1995 and 0.656 (± 0.034 SE) for 1997–2003 (see Kuningas et al., 2014), leading to an average across years of 0.606 (± 0.043 SE). In Period 2, the estimated proportion of identifiable individuals in the population varied from 0.687 (± 0.021 SE) in 2019 to 0.744 (± 0.023 SE) in 2015 (Table 10).

**TABLE 10:** Estimated proportion of identifiable killer whales in the population for each annual winter season

Year	Identifiable proportion (± SE)	Number of encounters
2012	0.716 (± 0.017)^a^	–
2013	0.716 (± 0.017)^a^	–
2014	0.716 (± 0.017)^a^	–
2015	0.744 (± 0.023)	18
2016	0.730 (± 0.016)	20
2017	0.716 (± 0.012)	19
2018	0.702 (± 0.014)	20
2019	0.687 (± 0.021)	31
Average/total	0.716 (± 0.017)	108

^a^ Few data were available for 2012–2014 so values for these years are given as the average over 2015–2019.

**FIGURE 9:**
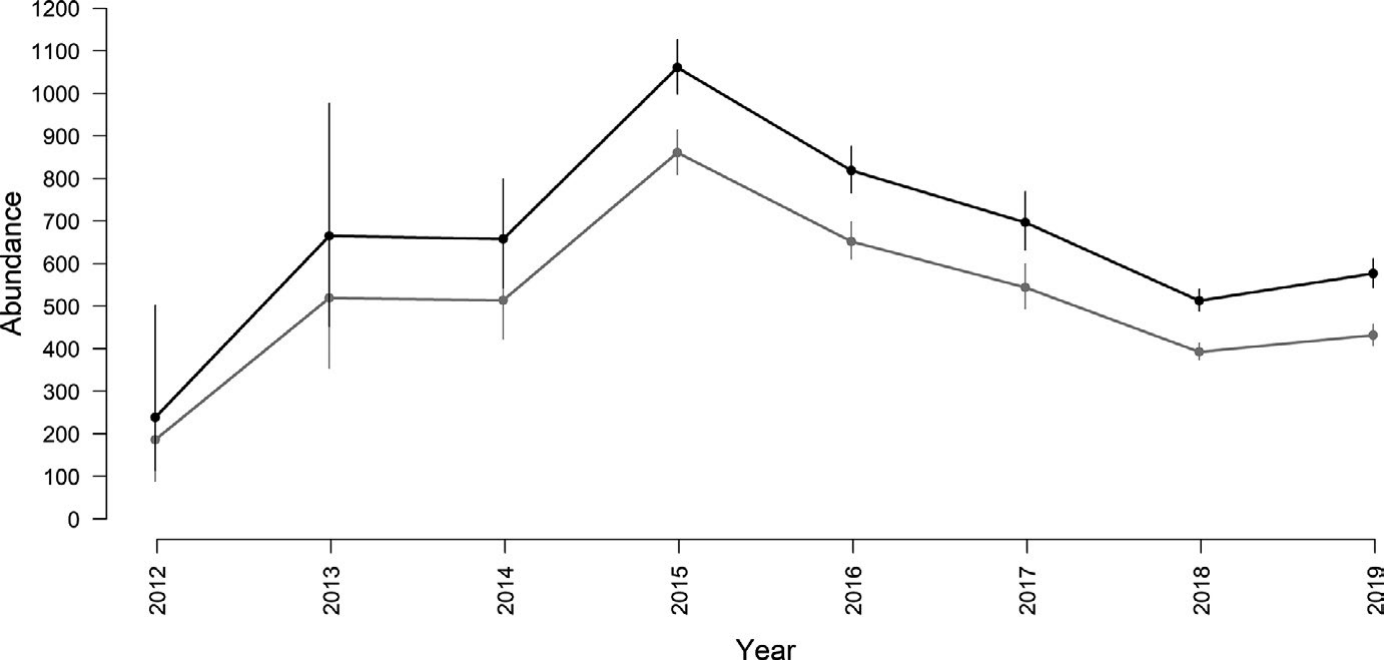
Model‐averaged estimates of the number of identifiable killer whales (gray) and total abundance (corrected for the proportion of identifiable individuals, black), with 95% CI, that used the study area during the winter months between 2012 and 2019, as estimated from the best‐supported robust design models listed in Table 7

Annual abundance, estimated from the RD models corrected for the proportion of identifiable individuals, peaked in 2015 at 1061 whales (95% CI: 999–1127) and dropped to 513 whales (95% CI: 488–540) in 2018 (Figure 9; Table S6). Large standard errors for estimates in 2012–2014 indicated low precision, most likely as a result of a relatively small number of identifications for these years (Figure S2). Annual abundance estimates obtained from POPAN models were comparable to those obtained from the RD, but less precise (Table S7).

Super‐population size in Period 1 (i.e., number of killer whales present in the study area at some point between 1988 and 2008), obtained by model‐averaging the most supported POPAN models (Table 8) and corrected for the average proportion of identifiable individuals across years (0.606, see above) was 886 (95% CI: 789–994). In Period 2, and accounting for an average proportion of identifiable individuals of 0.716 (see Table 10), super‐population size was 1894 (95% CI: 1806–1986). Other parameters estimated with POPAN models are given in the supplementary material (Figure S8).

## 4 DISCUSSION

Using a dataset on individual killer whales identified over three decades in northern Norway, we document survival rates and abundance estimates for the period 1988–2019. Models fitted to data from the two time periods 1988–2008 and 2012–2019 independently indicated changes in killer whales’ residency patterns, rather than a decrease in apparent survival suggested by a model fitted to the full time series.

### 4.1 Validation of method assumptions

To meet the assumption of correct mark recognition, only good quality photographs of reliably marked individuals were used (i.e., with multiple marks, see Figure S1), thus minimizing the risk of erroneous identifications. In addition, the photo‐identification work was carried out by the same experienced analysts throughout the study period, further minimizing inconsistencies during cataloguing and scoring of images (Urian et al., 2015). Lack of CJS model fit to the data was tested to facilitate model development and obtain robust estimates of apparent survival (Gimenez et al., 2018). In the full dataset, Test 2.CT (Table 4) indicated evidence of trap‐dependence in recapture probabilities, which was incorporated in the CJS models (Table 5). In our study, a behavioral trap response is unlikely because killer whales were photographically and not physically captured. Instead, heterogeneity in recapture probabilities could have been generated by sampling a restricted part of the range of the study population, by individual‐ or sex‐specific differences in behavior, or by nonrandom temporary emigration (Pradel & Sanz‐Aguilar, 2012). In most datasets, Test 3.SR indicated a transience effect that could have been caused by the presence of transient individuals (Pradel et al., 1997; Table 4). The strong support for time‐since‐marking models to account for transience (Tables 5 and 7) and the much lower estimates of apparent survival for transient individuals (see Section 3), confirmed that this was an effective way of dealing with this lack of fit of the CJS model.

### 4.2 Survival rates

In Period 1, estimates of apparent survival probability for adult killer whales of both sexes exceeded 0.98 and were higher for females than males throughout the study period (Figure 6a). These results are consistent with estimates from other killer whale populations (Esteban et al., 2016; Fearnbach et al., 2019; Jordaan et al., 2020; Olesiuk et al., 1990, 2005) and with previous analysis of similar data from northern Norway 1986–2003 (Kuningas et al., 2014). A contributing factor to the sex‐specific difference in survival is the extended postreproductive lifespan in females (Foster et al., 2012), which results in a longer mean life expectancy at birth for females than males (46 vs. 31 years in Northern Resident killer whales in British Columbia, Olesiuk et al., 2005). From 2012, after the NSS herring established new wintering grounds (Huse et al., 2010), apparent survival dropped for adult females (geometric mean: from 0.998 ± 0.002 SE to 0.955 ± 0.027 SE), and even more so for adult males (geometric mean: from 0.985 ± 0.009 SE to 0.864 ± 0.038 SE; Figure 6a). In such long‐lived species, a decrease in survival of this magnitude is highly unlikely to reflect natural variation in mortality but could be indicative of anthropogenic mortality. For example, survival estimates of killer whales at the Crozet Islands dropped from 0.99 to 0.92 (equivalent to an increase in apparent mortality rate from 1 to 8%) after the illegal Patagonian toothfish longline fisheries started in 1996 (Tixier et al., 2017). In this region, where killer whales depredate longlines as a feeding strategy, illegal vessels were reported to have used lethal means to repel the depredating whales, which led to an increased mortality risk (Poncelet et al., 2010; Tixier et al., 2017). In our study region, there is no evidence of increased mortality to explain the decline in apparent survival rates between the two periods. Thus, it is likely that the detected trend was a result of other features of the data.

Our analysis revealed important differences between the two periods. There was a substantially higher number of individual killer whales identified in 2012–2019 (*n* = 1032) compared to 1988–2008 (*n* = 352), despite Period 2 being much shorter. Notably, a much higher percentage of animals in Period 2 were transients (i.e., seen only once) compared to Period 1 (30% vs. 5%) (Figure 5). Lower recapture probabilities for the Low residency group (geometric mean: 0.239 ± 0.059 SE) compared to the High residency (geometric mean: 0.714 ± 0.050 SE) in Period 2 confirmed variation in residency patterns as a source of heterogeneity (Figure 8b). Not accounting for this heterogeneity in recapture probabilities when modeling the full time series caused the decline in apparent survival probabilities seen in Figure 6a. In Period 2, while the maximal apparent survival probabilities estimated for the High residency group were comparable to those estimated for Period 1, survival probabilities for the Low residency group were much lower (0.726 ± 0.074 SE; Figure 8a). This low apparent survival may result almost entirely from movement patterns of these animals modeled as permanent emigration. In support of this explanation, the drop in estimated apparent survival was greater for males (∆φ = 0.12) than females (∆φ = 0.04) in the full time series (Figure 6a), which likely is a consequence of most transients (75%) and individuals in the Low residency group (58%; Figure 7) being males. This is most likely an artifact in the data rather than emigration being more pronounced in males. It takes several years to reliably sex an individual as female (see Section 2), while adult males can be sexed upon first sighting based on their tall dorsal fin. Therefore, transient females would not have been identified up in the data. However, the short length of Period 2 (8 years) relative to the lifespan of a killer whale requires our results for this second period to be interpreted with caution. Even if it seems clear that true survival for the High residency group has not declined, we cannot entirely rule out a decline in true survival for the Low residency group.

### 4.3 Movement patterns and abundance

Robust design (RD) models for Period 2 indicated most support for random temporary emigration (Table 7). When random, and not Markovian, temporary emigration is not expected to bias survival estimates in CJS models, explaining the similarity in estimates of apparent survival from the RD and CJS models (Schaub et al., 2004). Five times more individuals showing low fidelity to the study area (Low residency group), compared to the 159 whales regularly seen (High residency group; Figure 7), further confirms movement in and out of the study area as an important characteristic of the study population.

Opportunistic photo‐identifications provided further corroboration of these movements; 71 of the individuals identified from winter surveys in the study area had also been photographed in other regions of the Norwegian coast and even offshore (Table 3; Figure 1). In 2012–2019, of the 523 (51%) identified individuals never seen again after the first year of capture (Figures 4 and 5), seven were photographed near Jan Mayen in summer in 2015 and 2016, confirming an offshore origin for at least some transient individuals. Notably, the single winter records of these whales were from 2015, the peak year of the number of transients and estimated abundance (Figures 5 and 9), and during which sampling covered open waters northwest of Andøya (Figure 1). Therefore, it appears likely that high herring abundance in coastal but open areas in some years, rather than in the inshore fjord system, attracted animals from elsewhere (including offshore) that had previously not been available to be sampled. This explanation is supported by the observation that 60% of the transients seen between 2012 and 2018 were identified at Andøya, even though this area contributed <30% of all captures for this period (EJ, unpublished data). The appearance of large numbers of humpback whales (and fin whales) at the newly established herring wintering grounds, which were not observed at former inshore locations in 1986–2006 (Jourdain & Vongraven, 2017), lends further support to this explanation.

As the photo‐identification study continues in these dynamic herring wintering grounds, sighting frequencies of individual killer whales are expected to vary over time. For example, a number of transients could be re‐identified in the future if coastal but open areas were to be surveyed again. Thus, what may appear as permanent emigration in the low estimates of apparent survival for the Low residency group in Period 2 could, in the future, contribute to temporary emigration in RD models.

Estimates of the number of killer whales that used the study area in a particular annual winter season varied considerably among years between 2012 and 2019 (Figure 9). Substantial fluctuations in killer whale abundance in the study area were also documented in 1990–2003 (Kuningas et al., 2014). As discussed above, this variability is likely linked to prey availability and associated killer whale movements. Killer whales are able to scout large areas to track the dynamic distribution of their herring prey and likely adjust their winter distribution accordingly (Similä & Stenersen, 2004). For example, Vengsøyfjord (surveyed in 2015–2016) and Kvænangen (surveyed in 2017–2019) held different herring year‐classes after the older year‐class started wintering offshore from 2017 (ICES, 2018). While some killer whales were still found in the fjords post‐2017, possibly owing to benefits from using the shallow bottom topography for hunting wintering herring (Nøttestad, 2002), others may have followed the larger, more profitable portion of the stock offshore. This hypothesis is supported by the lower abundance estimates in 2018–2019 compared to 2015–2017 (Figure 9).

Estimated annual killer whale abundance peaked at 731 individuals (95% CI: 505–1059, Kuningas et al., 2014) in Period 1, compared to 1061 (95% CI: 999–1127) in Period 2. Super‐population size estimated from POPAN models also increased from 911 (95% CI: 812–1022) in Period 1 to 1896 (95% CI: 1806–1991) in Period 2. As discussed above, it seems likely that this increase in abundance resulted from killer whales responding to the shifting of herring wintering grounds from the strictly inshore fjord system throughout Period 1 to both coastal (Vengsøyfjord and Kvænangen) and open waters (off Andøya) in Period 2 (Huse et al., 2010) (Figure 1).

A recent period of population growth also cannot be ruled out. Indeed, a demographic rebound following the end of the culling in 1982 (Øien, 1988) and the recovery of the NSS herring after a nearly total collapse in the late 1960s (Dragesund et al., 1997) may have been expected for killer whales in Norway. However, the combination of limited sampling and dynamic herring wintering grounds preclude the estimation of any meaningful trend in abundance, even in the sampled areas. In addition to a rebound to a precommercial fisheries ecosystem, killer whale population dynamics may be further affected by other ecological changes that are influenced by global warming. For example, the north‐eastern Atlantic mackerel, also a prey of killer whales in the study region (Nøttestad, Sivle, Krafft, Langard, et al., 2014), has greatly increased in biomass in the Norwegian Sea (Nøttestad, Utne, et al., 2015). Total super‐population size in 2012–2019 represented roughly 10 to 20% of killer whale abundance estimated from Norwegian shipboard surveys (Leonard & Øien, 2020a, 2020b). The large‐scale migration of the NSS herring, which couples offshore and coastal ecosystems, and the documented wide‐ranging capacities of killer whales in Norway (Table 5; Dietz et al., 2020; Similä & Stenersen, 2004; Vogel et al., 2021) suggest that the killer whales studied in northern Norway are part of a larger population of the Norwegian Sea and the wider Northeast Atlantic.

## 5 CONCLUSIONS

Re‐identification of individuals over multiple decades confirmed capture–recapture analysis as a suitable tool to monitor long‐term changes in the dynamics of killer whales in Norway. Our results show that the NSS herring remains a major ecological driver of killer whale dynamics in this region. We show that killer whales adjust their movement to shifting prey resources, indicating potential to adapt to rapidly changing marine ecosystems, as previously shown in other regions (e.g., Canadian Arctic, Ferguson et al., 2010). By shaping killer whale movement patterns, distributional prey shifts may also influence contact zones between killer whale groups, with possible implications for genetic population structure. Overall, killer whale abundance in northern Norwegian coastal waters shows an increase between 1988 and 2008 and post‐2012, although lower estimates in 2018–2019 may indicate a recent change. The variation in estimated annual abundance reflects variation in the proportion present in this area of an undefined population (or populations) in a larger geographical region. Our dataset is rare in its temporal extent and in its documentation of individual killer whales through a period characterized by marked ecosystem change. These data increase in value with each year that photo‐identification surveys are maintained. While the focus of this study was to explore population dynamics, this dataset is well suited to research questions at the individual level. Future studies should investigate how killer whales may be impacted by declining herring biomass (ICES, 2013, 2018), in the context of expected bottom‐up regulatory effects (Ford et al., 2010), and develop population models incorporating the effects of various stressors to inform conservation policy and any necessary management actions.

## CONFLICT OF INTEREST

Authors have no competing interests to declare.

## AUTHOR CONTRIBUTION

**Eve Marie Jourdain:** Conceptualization (lead); Data curation (equal); Formal analysis (lead); Funding acquisition (equal); Investigation (equal); Methodology (equal); Project administration (equal); Resources (equal); Software (equal); Visualization (equal); Writing‐original draft (lead); Writing‐review & editing (lead). **Tiffany Goh:** Formal analysis (equal); Methodology (equal); Software (equal); Visualization (equal); Writing‐review & editing (equal). **Sanna Kuningas:** Data curation (equal); Funding acquisition (equal); Investigation (equal); Resources (equal); Writing‐review & editing (equal). **Tiu Similä:** Data curation (equal); Funding acquisition (equal); Investigation (equal); Resources (equal); Writing‐review & editing (equal). **Dag Vongraven:** Data curation (equal); Funding acquisition (equal); Investigation (equal); Resources (equal); Writing‐review & editing (equal). **Richard Karoliussen:** Data curation (supporting); Funding acquisition (equal); Investigation (equal); Resources (equal); Writing‐review & editing (supporting). **Anna Bisther:** Data curation (equal); Funding acquisition (equal); Investigation (equal); Resources (equal); Writing‐review & editing (equal). **Philip Hammond:** Conceptualization (equal); Formal analysis (equal); Methodology (lead); Project administration (equal); Software (equal); Supervision (lead); Validation (lead); Writing‐original draft (equal); Writing‐review & editing (equal).

## Supporting information

Supinfo S1

## Data Availability

Capture histories of the killer whales photo‐identified in 1988–2019 in northern Norway, used to fit capture–recapture models, are available from Figshare data repository: https://doi.org/10.6084/m9.figshare.15112737.
