# Supplementary material for: Killer whale (Orcinus orca) population dynamics in response to a period of rapid ecosystem change in the eastern North Atlantic
Source: Ecol Evol. 2021 Nov 18;11(23):17289–306. doi: 10.1002/ece3.8364 (PMC8668809; doi:10.1002/ece3.8364)
Supplement: Supplementary file 1 — Supinfo S1 [file ECE3-11-17289-s001.docx]

***Appendix***

1. **Supplementary details on Methods**

**S1 Figure.** Photographs showing the criteria used to consider individual killer whales marked and reliably identifiable; individuals had to have at least one primary feature being (a) ≥ 3 scars on the saddle patch or (b) ≥ 2 nicks in the dorsal fin, or a combination of at least (c) two of the secondary features being <3 scars on the saddle patch, a distinctive pigmentation pattern of the saddle patch or a single nick in the dorsal fin; (d) Because killer whales with no nicks in the fin and showing scars only on the bottom section of the saddle patch would not be identifiable from photos showing only the upper 2/3 of the saddle patch, these individuals were not retained for analysis.


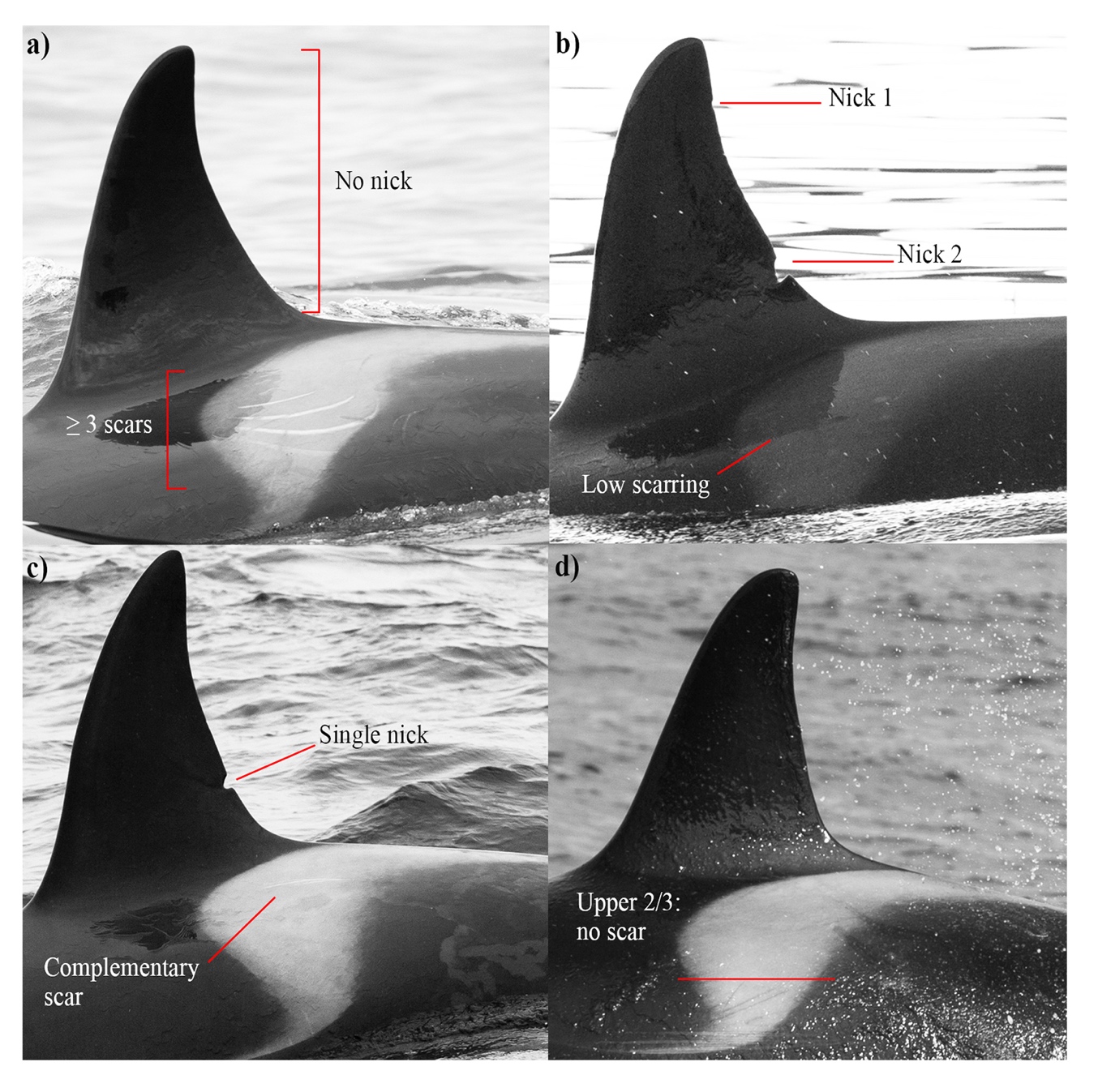


1. **Supplementary details on Results**

**S2 Figure.** Total number of observation days (bar plots) and total number of identifications (including resightings, line plot) from which the capture histories for the 1,236 killer whales in 1988-2019 used in this study were constructed. The vertical red solid line indicates the transition to Period 2 (2012-2019).


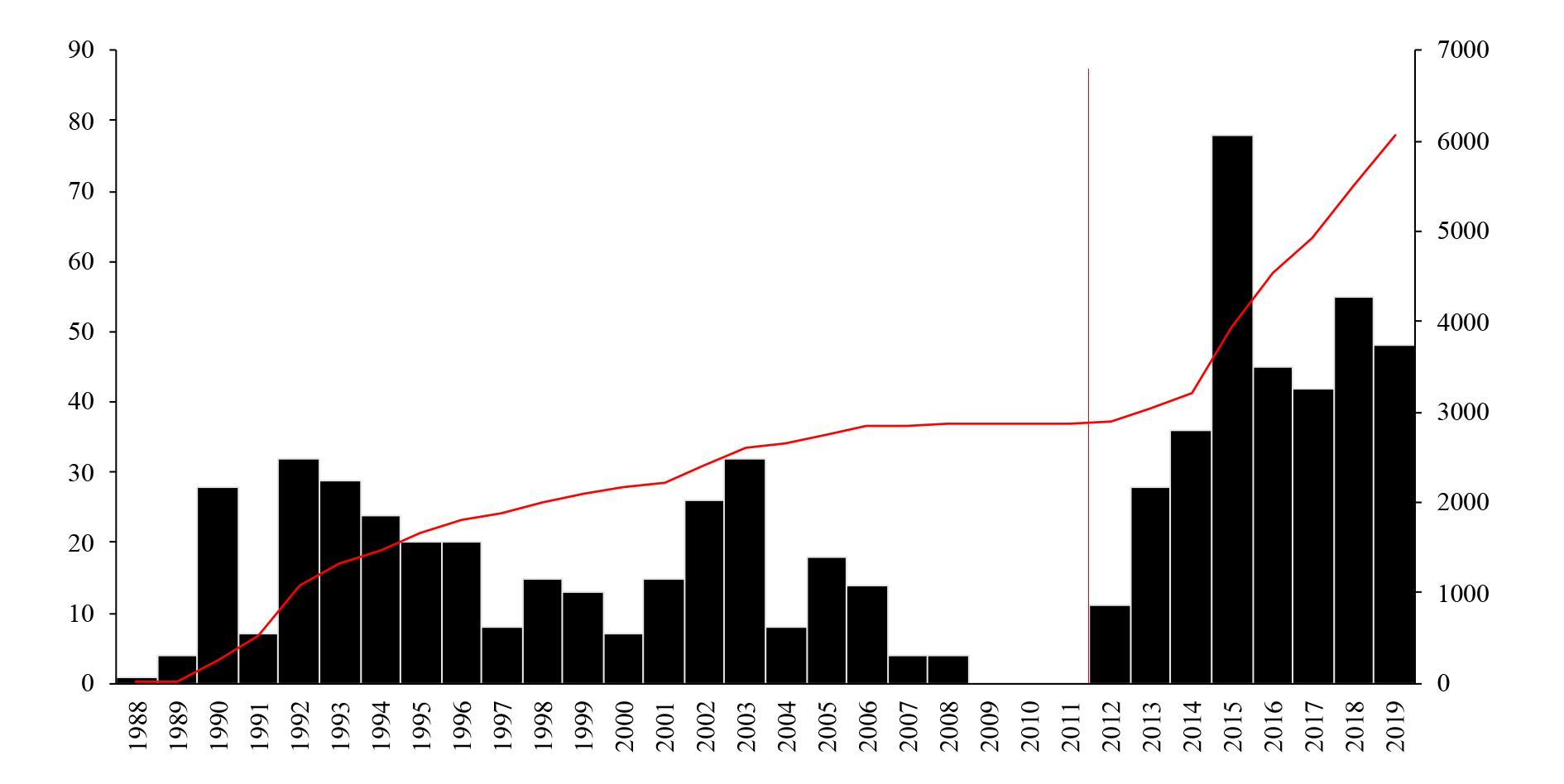


**S3 Table.** Matrix showing the number of distinct individuals identified in each year and the number of these individuals recaptured in the subsequent years.

| Year | Captured | 1989 | 1990 | 1991 | 1992 | 1993 | 1994 | 1995 | 1996 | 1997 | 1998 | 1999 | 2000 | 2001 | 2002 | 2003 | 2004 | 2005 | 2006 | 2007 | 2008 | 2012 | 2013 | 2014 | 2015 | 2016 | 2017 | 2018 | 2019 |
| --- | --- | --- | --- | --- | --- | --- | --- | --- | --- | --- | --- | --- | --- | --- | --- | --- | --- | --- | --- | --- | --- | --- | --- | --- | --- | --- | --- | --- | --- |
| 1988 | 7 | 4 | 6 | 6 | 6 | 1 | 0 | 1 | 2 | 0 | 4 | 4 | 1 | 1 | 1 | 1 | 1 | 3 | 0 | 0 | 0 | 0 | 0 | 0 | 1 | 3 | 0 | 1 | 0 |
| 1989 | 10 |  | 9 | 7 | 6 | 4 | 4 | 5 | 5 | 3 | 6 | 7 | 3 | 1 | 3 | 2 | 3 | 4 | 0 | 7 | 7 | 0 | 1 | 2 | 4 | 5 | 1 | 1 | 3 |
| 1990 | 107 |  |  | 59 | 87 | 57 | 40 | 36 | 44 | 24 | 40 | 59 | 26 | 13 | 36 | 36 | 11 | 20 | 8 | 0 | 0 | 0 | 3 | 7 | 19 | 21 | 9 | 11 | 15 |
| 1991 | 110 |  |  |  | 86 | 58 | 35 | 42 | 28 | 21 | 38 | 37 | 30 | 12 | 25 | 28 | 7 | 19 | 12 | 0 | 0 | 0 | 5 | 7 | 19 | 20 | 11 | 15 | 15 |
| 1992 | 169 |  |  |  |  | 80 | 55 | 63 | 57 | 33 | 52 | 55 | 34 | 18 | 46 | 48 | 12 | 28 | 16 | 0 | 0 | 0 | 4 | 10 | 30 | 31 | 15 | 20 | 23 |
| 1993 | 102 |  |  |  |  |  | 46 | 42 | 38 | 21 | 42 | 43 | 34 | 11 | 31 | 34 | 8 | 17 | 12 | 0 | 0 | 0 | 5 | 8 | 19 | 21 | 11 | 17 | 20 |
| 1994 | 81 |  |  |  |  |  |  | 31 | 31 | 17 | 48 | 33 | 24 | 8 | 24 | 27 | 10 | 14 | 10 | 0 | 0 | 0 | 2 | 7 | 19 | 21 | 11 | 14 | 15 |
| 1995 | 82 |  |  |  |  |  |  |  | 29 | 23 | 29 | 29 | 23 | 11 | 25 | 22 | 5 | 17 | 9 | 0 | 0 | 0 | 5 | 6 | 19 | 20 | 10 | 11 | 15 |
| 1996 | 72 |  |  |  |  |  |  |  |  | 19 | 25 | 33 | 20 | 9 | 29 | 28 | 6 | 16 | 7 | 0 | 0 | 1 | 3 | 7 | 20 | 16 | 12 | 18 | 18 |
| 1997 | 37 |  |  |  |  |  |  |  |  |  | 19 | 19 | 12 | 5 | 14 | 14 | 3 | 6 | 3 | 0 | 0 | 0 | 2 | 3 | 11 | 8 | 2 | 4 | 5 |
| 1998 | 72 |  |  |  |  |  |  |  |  |  |  | 35 | 21 | 10 | 24 | 28 | 10 | 20 | 3 | 0 | 0 | 1 | 3 | 7 | 23 | 28 | 14 | 20 | 17 |
| 1999 | 64 |  |  |  |  |  |  |  |  |  |  |  | 21 | 10 | 27 | 31 | 10 | 17 | 3 | 0 | 0 | 0 | 1 | 5 | 13 | 15 | 3 | 5 | 7 |
| 2000 | 39 |  |  |  |  |  |  |  |  |  |  |  |  | 4 | 14 | 12 | 3 | 7 | 3 | 0 | 0 | 0 | 2 | 3 | 10 | 9 | 4 | 7 | 9 |
| 2001 | 28 |  |  |  |  |  |  |  |  |  |  |  |  |  | 7 | 14 | 1 | 5 | 5 | 0 | 0 | 0 | 2 | 2 | 6 | 3 | 4 | 5 | 4 |
| 2002 | 86 |  |  |  |  |  |  |  |  |  |  |  |  |  |  | 40 | 8 | 19 | 11 | 0 | 0 | 0 | 7 | 7 | 29 | 23 | 15 | 21 | 19 |
| 2003 | 89 |  |  |  |  |  |  |  |  |  |  |  |  |  |  |  | 12 | 20 | 9 | 0 | 0 | 1 | 5 | 4 | 36 | 20 | 17 | 22 | 15 |
| 2004 | 24 |  |  |  |  |  |  |  |  |  |  |  |  |  |  |  |  | 11 | 2 | 0 | 0 | 0 | 1 | 3 | 11 | 11 | 8 | 7 | 7 |
| 2005 | 71 |  |  |  |  |  |  |  |  |  |  |  |  |  |  |  |  |  | 7 | 2 | 1 | 1 | 2 | 5 | 28 | 23 | 18 | 25 | 22 |
| 2006 | 48 |  |  |  |  |  |  |  |  |  |  |  |  |  |  |  |  |  |  | 2 | 1 | 0 | 5 | 10 | 20 | 20 | 14 | 21 | 20 |
| 2007 | 3 |  |  |  |  |  |  |  |  |  |  |  |  |  |  |  |  |  |  |  | 1 | 0 | 0 | 1 | 0 | 1 | 2 | 3 | 2 |
| 2008 | 6 |  |  |  |  |  |  |  |  |  |  |  |  |  |  |  |  |  |  |  |  | 0 | 0 | 2 | 1 | 3 | 3 | 3 | 3 |
| 2012 | 36 |  |  |  |  |  |  |  |  |  |  |  |  |  |  |  |  |  |  |  |  |  | 1 | 6 | 8 | 2 | 4 | 2 | 0 |
| 2013 | 102 |  |  |  |  |  |  |  |  |  |  |  |  |  |  |  |  |  |  |  |  |  |  | 26 | 38 | 25 | 14 | 15 | 15 |
| 2014 | 142 |  |  |  |  |  |  |  |  |  |  |  |  |  |  |  |  |  |  |  |  |  |  |  | 61 | 33 | 18 | 27 | 26 |
| 2015 | 474 |  |  |  |  |  |  |  |  |  |  |  |  |  |  |  |  |  |  |  |  |  |  |  |  | 163 | 111 | 125 | 118 |
| 2016 | 381 |  |  |  |  |  |  |  |  |  |  |  |  |  |  |  |  |  |  |  |  |  |  |  |  |  | 140 | 136 | 128 |
| 2017 | 266 |  |  |  |  |  |  |  |  |  |  |  |  |  |  |  |  |  |  |  |  |  |  |  |  |  |  | 122 | 102 |
| 2018 | 286 |  |  |  |  |  |  |  |  |  |  |  |  |  |  |  |  |  |  |  |  |  |  |  |  |  |  |  | 156 |
| 2019 | 290 |  |  |  |  |  |  |  |  |  |  |  |  |  |  |  |  |  |  |  |  |  |  |  |  |  |  |  |  |

**S4 Table.** Number of individuals identified as adult males (M), adult females (F) and Unknowns (U) in all three datasets (Period 1: 1988-2008, Period 2: 2012-2019 and full time series: 1988-2019).

|  |  | Total | M | F | U |
| --- | --- | --- | --- | --- | --- |
| Period 1 | No. individuals (IDs) | 352 | 218 | 61 | 73 |
|  | No. captures | 1,307 | 811 | 244 | 252 |
| Period 2 | No. individuals (IDs) | 1,032 | 589 | 238 | 205 |
|  | No. captures | 1,977 | 1,199 | 500 | 278 |
| Full series | No. individuals (IDs) | 1,236 | 719 | 241 | 276 |
|  | No. captures | 3,284 | 2,010 | 744 | 530 |

**S5 Figure.** Non-sex-specific model-averaged probabilities of a) apparent survival between years and of b) capture for each secondary sampling occasion, with 95% CI, as estimated from the best-supported Robust Design models listed in Table 7.


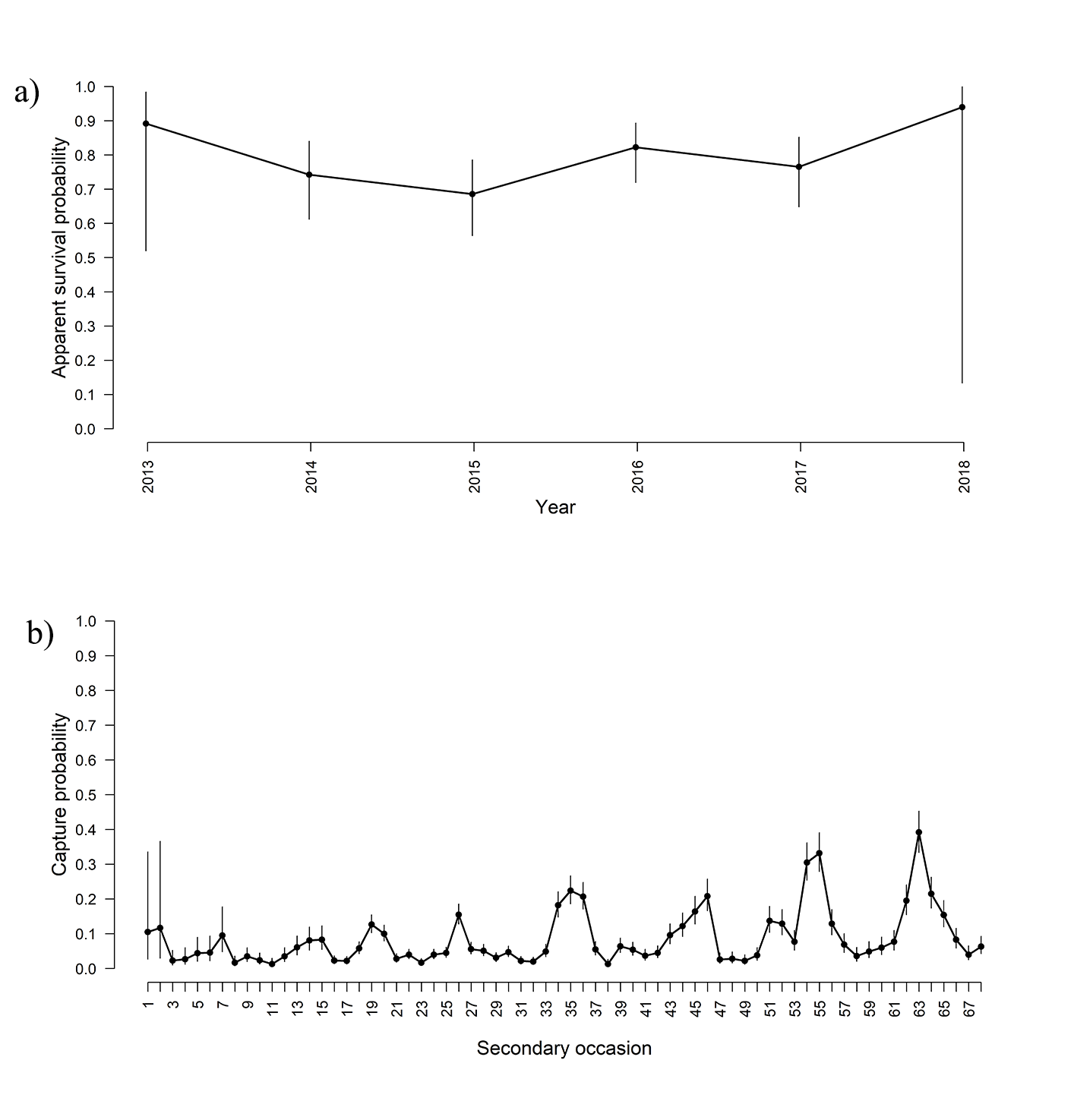


**S6 Table.** Model-averaged abundance estimates, *N*, for each annual winter season in 2012-2019 as obtained from the Robust Design models listed in Table 7, and estimates of total population abundance, *N_total_*, obtained by dividing *N* by the proportion identifiable in the population from Table 10.

|  | Identifiable population | | | Total population | | |
| --- | --- | --- | --- | --- | --- | --- |
|  | *N* | SE | 95% CI | *N_total_* | SE | 95% CI |
| 2012 | 171 | 107 | 81-360 | 239 | 150 | 114-502 |
| 2013 | 476 | 145 | 324-700 | 665 | 203 | 453-977 |
| 2014 | 471 | 71 | 389-571 | 658 | 99 | 542-799 |
| 2015 | 789 | 37 | 743-838 | 1,061 | 50 | 999-1,127 |
| 2016 | 598 | 31 | 559-639 | 819 | 42 | 766-876 |
| 2017 | 499 | 38 | 453-550 | 697 | 53 | 632-769 |
| 2018 | 360 | 14 | 342-379 | 513 | 20 | 488-540 |
| 2019 | 396 | 18 | 373-420 | 577 | 26 | 544-611 |

**S7 Table.** Model-averaged abundance estimates, *N*, for each annual winter season in Period 1 (1988-2008) and in Period 2 (2012-2019) as obtained from POPAN models listed in Tables 8 and 9, and estimates of total population abundance, *N_total_*, obtained by dividing *N* by the proportion identifiable in the population from Table 10. Abundance was not estimated for 2012.

|  | Identifiable population | | | Total population | | |
| --- | --- | --- | --- | --- | --- | --- |
|  | *N* | SE | 95% CI | *N_total_* | SE | 95% CI |
| Period 1 |  |  |  |  |  |  |
| 1988 | 234 | 40 | 155-312 | 386 | 66 | 309-480 |
| 1989 | 231 | 35 | 162-299 | 381 | 58 | 313-463 |
| 1990 | 225 | 30 | 166-285 | 372 | 50 | 313-442 |
| 1991 | 221 | 26 | 169-272 | 364 | 43 | 312-424 |
| 1992 | 216 | 23 | 171-261 | 357 | 38 | 311-409 |
| 1993 | 212 | 20 | 172-252 | 351 | 34 | 310-397 |
| 1994 | 209 | 19 | 172-246 | 345 | 31 | 307-388 |
| 1995 | 207 | 18 | 171-243 | 341 | 30 | 304-383 |
| 1996 | 205 | 19 | 168-241 | 338 | 31 | 301-380 |
| 1997 | 204 | 19 | 166-242 | 337 | 32 | 298-381 |
| 1998 | 204 | 20 | 165-244 | 337 | 33 | 297-383 |
| 1999 | 206 | 21 | 164-247 | 339 | 35 | 297-388 |
| 2000 | 208 | 22 | 165-251 | 344 | 36 | 300-394 |
| 2001 | 213 | 23 | 168-257 | 351 | 38 | 306-403 |
| 2002 | 219 | 24 | 172-265 | 361 | 39 | 314-415 |
| 2003 | 227 | 25 | 177-277 | 375 | 42 | 325-433 |
| 2004 | 238 | 30 | 180-296 | 393 | 49 | 335-461 |
| 2005 | 252 | 37 | 179-325 | 416 | 62 | 344-503 |
| 2006 | 269 | 50 | 171-368 | 444 | 83 | 350-564 |
| 2007 | 291 | 69 | 155-426 | 480 | 114 | 354-649 |
| 2008 | 317 | 95 | 130-504 | 523 | 157 | 357-765 |
| Period 2 |  |  |  |  |  |  |
| 2012 | - | - | - | - | - | - |
| 2013 | 611 | 124 | 368-854 | 854 | 173 | 659-1,107 |
| 2014 | 672 | 239 | 203-1,141 | 939 | 334 | 601-1,466 |
| 2015 | 808 | 80 | 650-966 | 1,086 | 108 | 956-1,234 |
| 2016 | 700 | 69 | 565-836 | 959 | 95 | 844-1,089 |
| 2017 | 596 | 40 | 517-674 | 832 | 56 | 763-908 |
| 2018 | 473 | 36 | 403-544 | 674 | 51 | 611-744 |
| 2019 | 355 | 120 | 120-590 | 517 | 175 | 338-791 |

**S8 Figure.** Model-averaged capture (black) and recruitment (*pent*, grey) probabilities with 95% CI as obtained from the best-supported POPAN models for a) Period 1 (1988-2008, models listed in Table 8) and Period 2 (2012-2019, models listed in Table 9).

**
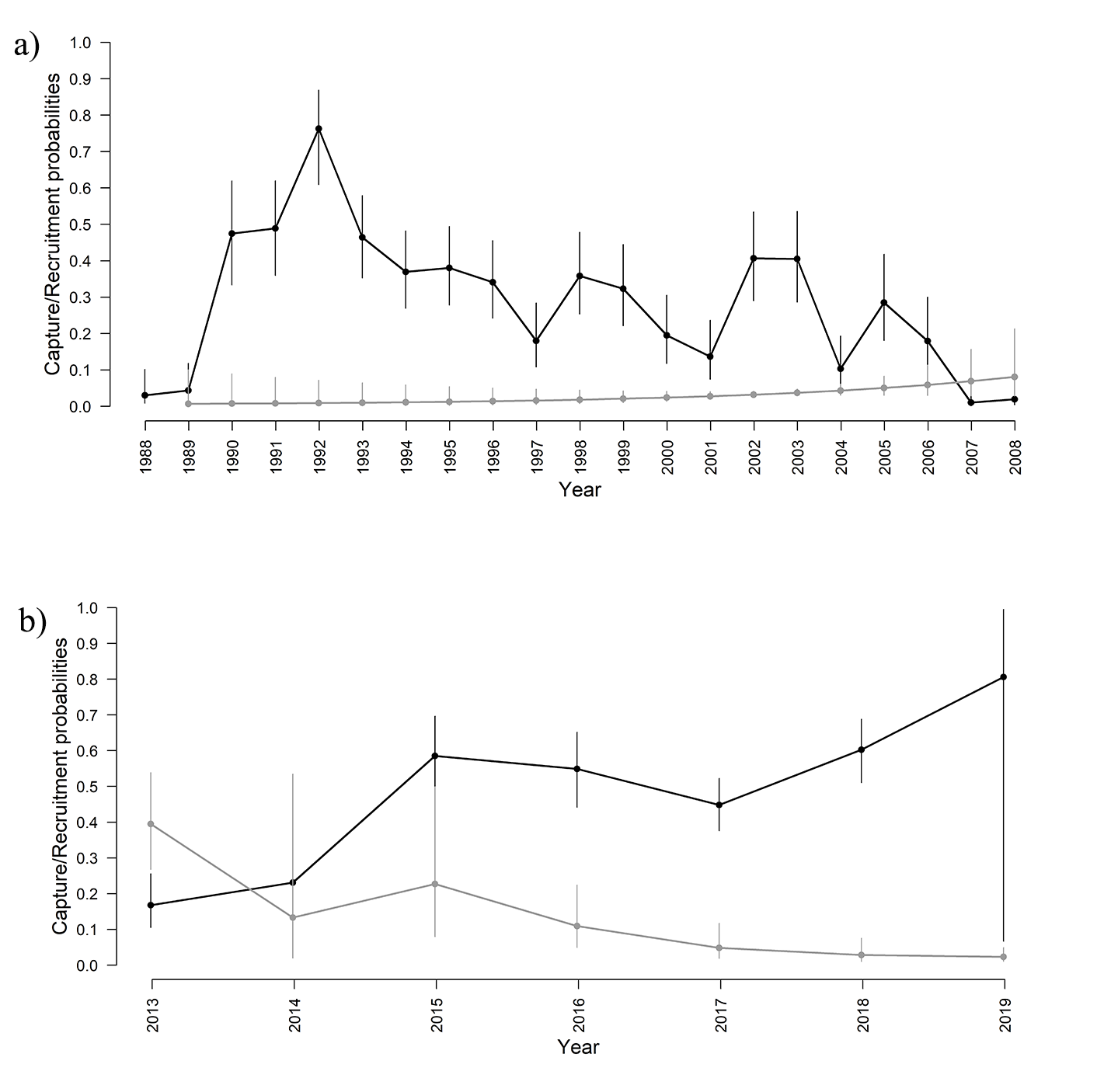
**
